# A multivariate ecogeographic analysis of macaque craniodental variation

**DOI:** 10.1002/ajpa.23439

**Published:** 2018-02-15

**Authors:** Nicole D. S. Grunstra, Philipp Mitteroecker, Robert A. Foley

**Affiliations:** ^1^ Leverhulme Centre for Human Evolutionary Studies, Department of Archaeology and Anthropology University of Cambridge, The Henry Wellcome Building Cambridge CB2 1QH United Kingdom; ^2^ Department of Theoretical Biology University of Vienna Vienna, 1090 Austria

**Keywords:** environmental gradient, phylogeny, rainfall, spatial analysis, temperature

## Abstract

**Objectives:**

To infer the ecogeographic conditions that underlie the evolutionary diversification of macaques, we investigated the within‐ and between‐species relationships of craniodental dimensions, geography, and environment in extant macaque species. We studied evolutionary processes by contrasting macroevolutionary patterns, phylogeny, and within‐species associations.

**Materials and Methods:**

Sixty‐three linear measurements of the permanent dentition and skull along with data about climate, ecology (environment), and spatial geography were collected for 711 specimens of 12 macaque species and analyzed by a multivariate approach. Phylogenetic two‐block partial least squares was used to identify patterns of covariance between craniodental and environmental variation. Phylogenetic reduced rank regression was employed to analyze spatial clines in morphological variation.

**Results:**

Between‐species associations consisted of two distinct multivariate patterns. The first represents overall craniodental size and is negatively associated with temperature and habitat, but positively with latitude. The second pattern shows an antero‐posterior tooth size contrast related to diet, rainfall, and habitat productivity. After controlling for phylogeny, however, the latter dimension was diminished. Within‐species analyses neither revealed significant association between morphology, environment, and geography, nor evidence of isolation by distance.

**Discussion:**

We found evidence for environmental adaptation in macaque body and craniodental size, primarily driven by selection for thermoregulation. This pattern cannot be explained by the within‐species pattern, indicating an evolved genetic basis for the between‐species relationship. The dietary signal in relative tooth size, by contrast, can largely be explained by phylogeny. This cautions against adaptive interpretations of phenotype–environment associations when phylogeny is not explicitly modelled.

## 1 INTRODUCTION

A major goal of evolutionary biology is to reveal how the phenotype has evolved in response to its ecological and geographic environment. Many studies have shown that phenotypic variation in primates carries both geographic (i.e., the effect of distance and isolation) and environmental (i.e., the effect of ecological factors) signals (Cardini and Elton, 2009; Cardini et al., 2007; Dunn et al., 2013; Frost et al., 2003; Ito et al., 2014; Kamilar et al., 2012; Lehman et al., 2005; Meloro et al., 2014; Viguier, 2004). However, the exact patterns often differ between taxa living in different environments, exposed to different selective forces, and living in different geographic contexts. But similar ecogeographic patterns can nonetheless result from different evolutionary processes (Meiri, 2011). For example, Bergmann's rule describes a general tendency for endotherms to have larger body sizes at higher latitudes, within (Ashton et al., 2000; Mayr, 1956; Rensch, 1938) as well as between species (Bergmann, 1847; Millien et al., 2006). Explanations of this pattern typically invoke a thermoregulatory effect of cold temperatures on animal body size at higher latitudes (Mayr, 1956; Meiri and Dayan, 2003), but in some taxa rainfall may better explain the relationship between size and latitude (Ashton et al., 2000; Millien et al., 2006), including some nonhuman primates (Cardini et al., 2007; Frost et al., 2003). Such contrasting ecological correlates of Bergmann's rule indicate that different selective forces may give rise to the same pattern (Meiri and Dayan, 2003).

In a taxon‐wide study of Bergmann's rule in primates, a positive relationship between latitude and body mass was found among non‐Malagasy primates (Harcourt and Schreier, 2009). However, at a lower taxonomic level, and after controlling for phylogeny, the pattern only persisted in macaque species living on the Asian continental shelf (Harcourt and Schreier, 2009). Furthermore, size gradients that correlate not with latitude but with longitude have been retrieved for cranial size within several African cercopithecid primates (vervet monkeys: Cardini et al., 2007; red colobus monkeys: Cardini and Elton, 2009; greater spot‐nosed and blue monkey: Cardini et al., 2010; and baboons: Dunn et al., 2013). By contrast, cranial shape varies more strongly along a latitudinal than a longitudinal gradient between several Neotropical species of howler and capuchin monkeys (Cáceres et al., 2014; Meloro et al., 2014), and between some (but not all) macaques (Ito et al., 2014). Bergmann's rule is, however, a special case of the more general principle of ecomorphology; Bergmann's rule concerns temperature and body size, but evolutionary ecomorphology investigates multiple interactions between environmental parameters and organismal shape and size.

To date, only a limited number of primate studies have included multiple climate and ecological variables (e.g., Cardini et al., 2007; Harvati and Weaver, 2006; Kamilar et al., 2012; Meloro et al., 2014; Viguier, 2004). From these studies, a mixed pattern of the environmental correlates of morphological size and shape variation in primates emerges. Rainfall and other humidity measures, as indicators of habitat productivity, are relevant in explaining cranial variation in vervet monkeys (Cardini et al., 2007), some Malagasy sifakas (Lehman et al., 2005), and lemurs (Viguier, 2004). In New World capuchin monkeys, however, both rainfall and temperature are important climatic predictors of skull shape (Cáceres et al., 2014). A recent environmental analysis of Malagasy strepsirrhine body mass revealed that diet and climate were weak predictors of body size, but that there was a strong phylogenetic effect (Kamilar et al., 2012). In modern humans, signals of population history in cranial variation have been found to be stronger than, or even drive, climatic signatures, highlighting the role of population structure and genetic drift (Betti et al., 2010; Harvati and Weaver, 2006; Roseman and Auerbach, 2015). It is becoming increasingly apparent that primate evolution, within and between species, has been characterized by a complex interplay of different selective forces and neutral processes.

Here, we carry out, to our knowledge, the first detailed multivariate analysis of craniodental dimensions and their relation to geographic distribution, climate and species' ecology in the radiation of macaques (Cercopithecidae: *Macaca*) in a phylogenetic framework. Macaques are an interesting taxon because they diversified widely and rapidly during times of considerable environmental change in the Pliocene and Pleistocene (Abegg and Thierry, 2002; Brandon‐Jones, 1996; Delson, 1980), and because they continue to occupy a range of different habitats across southern, central, eastern, and insular Asia and North Africa today (Fooden, 1982).

The main focus of our study is to elucidate the role of the environment in phenotypic and taxonomic diversification at the macroevolutionary level by studying between‐species variation within a given phylogeny. Our choice of phenotype is the dentition and associated cranial structures, as teeth are minimally plastic and are therefore likely to carry stronger evolutionary signals than other phenotypes. We compare the interspecific patterns to the intraspecific patterns in order to infer evolutionary processes. Macaques are well known for occupying a great diversity of environments in terms of climate, geographical distribution, and resource exploitation. Therefore, we also investigate here how multiple and diverse aspects of the environment may have impacted diversification of macaques, by specifically studying how they interact in their association to macaque craniodental variation. We expect the effect of climate on macaque thermoregulation and the influence of resource exploitation on the dentition to underlie the functional links between macaque craniodental morphology and the environment. Specifically, we test if the Bergmann effect is reflected in dental patterns, as expected on the basis of a Bergmannian trend in macaque body mass (Harcourt and Schreier, 2009), and explore its ecological and environmental correlates. Furthermore, we expect that dental patterns, such as relative incisor and molar size, covary with food type and thus in turn with climate and geography.

Instead of studying associations between single selected environmental and phenotypic variables, we employ a fully multivariate approach. We search for multivariate patters that jointly underlie the association between all ecological, geographic, and morphological variables. This exploratory approach requires careful interpretation but allows us to identify the actual complexity (number of independent factors) within this association, without prior specification of the number of variables considered. This approach also allows us to investigate the role of a wider range of potentially relevant variables not (often) explored elsewhere. While partial least squares analysis—our method of choice to relate ecological to morphometric variables—has been used in the literature already, the application of reduced rank regression to identify multivariate morphological clines is (to our knowledge) novel.

The effect of species' phylogenetic relatedness on observed relationships is often either not modelled or simply removed as part of the analysis. Here, we examine in detail how the patterns and magnitude of covariation between phenotype and ecogeography in macaques are influenced by phylogeny. In other words, we estimate the extent to which observed phenotype–environment associations in *Macaca* are consistent with shared ancestry in order to gauge adaptive interpretations such as evolutionary convergence. To this end, we carry out the between‐species analyses both with and without phylogenetic correction.

## 2 MATERIALS AND METHODS

### 2.1 Morphometric and contextual data

Twelve macaque species were used in this study (Table 1), selected to capture the ecogeographic diversity across the genus in combination with practical considerations regarding their availability in museum collections. A total of 711 specimens (Table 1) were measured at the following institutions: The National Museum of Natural History (Washington, DC), Senckenberg Forschungsinstitut und Naturmuseum Frankfurt, Naturalis Biodiversity Center (Leiden), Natural History Museum (London), Muséum National d'Histoire Naturelle (Paris), Museum für Naturkunde (Berlin), The Royal College of Surgeons of England (London), and the Naturhistorisches Museum Wien (Vienna). Preference was given to wild‐caught and wild‐shot specimens, although occasionally captive specimens or those without provenience data were included to obtain acceptable sample sizes.

**Table 1:** Macaque species included in this study, including their geographical distribution (Abegg & Thierry, 2002), as well as the number of specimens measured for this study (includes subadult individuals). Classification as per Groves (2005)

Binomial name	Common name	Distribution	Males (*N*)	Females (*N*)	Total (*N*)
*M. assamensis*	Assamese macaque	Continental Southeast Asia	13	6	19
*M. cyclopis*	Taiwanese macaque	Taiwan	7	11	18
*M. fascicularis*	Long‐tailed macaque	Indochinese peninsula, Indonesia, Philippines	51	41	92
*M. fuscata*	Japanese macaque	Japan	24	20	44
*M. maura*	Moor macaque	Southwest Sulawesi	34	20	54
*M. mulatta*	Rhesus macaque	Continental South and East Asia	33	43	76
*M. nemestrina*	Southern pigtailed macaque	Malay peninsula, Sumatra, Borneo	39	23	62
*M. nigra*	Crested macaque	North Sulawesi	37	37	74
*M. radiata*	Bonnet macaque	South and West India	46	33	79
*M. silenus*	Lion‐tailed macaque	Southwest India	24	21	45
*M. sinica*	Toque macaque	Sri Lanka	40	35	75
*M. sylvanus*	Barbary macaque	Algeria, Morocco	36	37	73
**Total**			**384**	**327**	**711**

A total of 46 linear measurements of tooth size were taken on the permanent maxillary and mandibular teeth. Tooth lengths and breadths were measured for all teeth, complemented by tooth height for the anterior dentition, following a standard approach (e.g., Swindler, 2002). Teeth on the right side of the jaw were measured where possible; broken or missing teeth were substituted by the left antimere. The key to tooth variable names is described in Table 2, and tooth measurement protocols can be found in Tables S1–S3 in the Supporting Information. In this article, the incisors and the canines are referred to as the anterior dentition, and the premolars and molars as the posterior (or postcanine) dentition. An additional 17 cranial, maxillary, and mandibular measurements were taken to represent the structure that houses the dentition and to provide a “morphological context” for the dental variables (Table 3). These measurements were only recorded on adult specimens showing full eruption of their third molars to minimize ontogenetic variation. Because the skeletal and dental measurements showed the same results separately as they did combined, we only present the results for the combined phenotypic dataset.

**Table 2:** Key to variable abbreviations of the 46 tooth size measurements (length and width of all teeth, plus height for the incisors and canines). For example, the mesiodistal length of the upper first (central) incisor is abbreviated as UI1MD, the anterior width of the lower third molar as LM3AW

Prefix	U = upper (maxillary), L = lower (mandibular).
Tooth class	I = incisor, C = canine, P = premolar, M = molar.
Tooth position	incisors (1 = central, 2 = lateral), canines (number n/a), premolars (3 = mesial, 4 = distal), molars (1 = mesial, 2 = central, 3 = distal).
Dimension	MD = mesiodistal length of incisors and canines, LL = labiolingual width of incisors, H = height of incisors and canines, BL = buccolingual width of canines, L = mesiodistal length of premolars and molars (except P_3_), OL and TL = occlusal and total length (includes the sectorial crest) of the lower third premolar (P_3_), W = buccolingual width of premolars, AW and PW = anterior and posterior buccolingual width of molars.

**Table 3:** Definitions of cranial, maxillary and mandibular variables and their measurement descriptions

Variable name	Measurement description
CALV	Calvarium length; distance from nasion to occipital protuberance.
PALWID	Palate width; distance between left M^2^ (lingual point) and right M^2^ (lingual point).
pr‐alv	Palate length; distance from prosthion to alveolon.
ba‐pr	Distance from basion to prosthion.
MUZL	Muzzle length; distance from mesial orbital margin to alveolar margin at I^1^.
UIAW	Tooth row length of upper incisors; measured from distal border of left I^2^ to distal border of right I^2^ (at the cemento‐enamel junction (CEJ)).
LIAW	Tooth row length of lower incisors; as per UIAW.
UBCB	Bi‐canine breadth of upper canines; measured from the buccal surface of the left canine to the buccal surface of the right canine (at the CEJ).
LBCB	Bi‐canine breadth of lower canines; as per UBCB.
UpcRow	Upper postcanine tooth row length; distance from mesial border of P^3^ to distal border of M^3^ (measured at CEJ).
LpcRow	Lower postcanine tooth row length; as per UpcRow.
Uecm‐ecm	Maximum width of upper dental arcade; distance from left to right ectomolare (measured on the alveolar bone at M^2^).
Lecm‐ecm	Maximum width of lower dental arcade; as per Uecm‐ecm.
CONM1	Distance from tip of mandibular condyle to mesial border of M_1_.
Mand_height	Mandible height; measured at mesial M_2_ (on the alveolar bone) at a right angle down to inferior surface of mandibular corpus.
mand_thick	Mandible thickness; measured from medioposterior mandibular symphysis to surface point on anterior mandibular corpus, at a right angle to dental arcade.
go_go	Mandibular width; distance from left gonion to right gonion.

All measurements were taken by the same person (NDSG) with digital dental callipers (Mitutoyo, 573 series, Kawasaki, Japan) with an accuracy of 0.01 mm. Intraobserver mean measurement error (derived from a subsample of 50 specimens, with two replicates for each measurement) was 0.18 mm (an average of 2.2% error of the mean) and the intraclass correlation coefficient was 0.99. All morphometric data used in this study are available at doi:10.5281/zenodo.182699.

#### 2.1.1 Between‐species analysis

For the interspecific analysis, species means were computed from the phenotypic data by taking the arithmetic mean of the female and male means of each species. We pool male and female phenotypes in this analysis as the environmental and geographic variables are the same for both sexes, and strong sex‐specific interaction effects between ecogeography and craniodental variation seem unlikely.

Contextual data of macaque ecogeography were collected from published sources. Henceforth, we use “ecogeography” to refer to geographic, environmental, and ecological parameters relating to macaque spatial distribution, climate, as well as habitat and dietary ecology. We use variables that represent average environmental conditions (e.g., mean temperature, minimum rainfall, median altitude, mean habitat productivity) and variables that reflect environmental heterogeneity itself (e.g., range size variables, climatic seasonality indices, and habitat and dietary breadth). All ecogeographic parameters are described below.

Spatial geography is represented by latitude and longitude, taken as the coordinates of the central point in each species' geographic range along with geographic range size and latitudinal and longitudinal ranges. The range variables and the central coordinates as well as actual evapotranspiration rate (AET; a measure of habitat productivity) were obtained from the PanTHERIA database (Jones et al., 2009). As several macaque species have successfully dispersed to insular Southeast and East Asia, species were assigned to one of the following categories: (1) island(s) only, (2) mixed, (3) continental mainland only. Data can be found in Supporting Information Table S4.

Climate variables were selected from among the bioclimatic variables in the WorldClim database (Hijmans et al., 2005) and data were retrieved at a resolution of 2.5 arc‐minutes. The variables used in this study are common climatic measures, namely mean, minimum and maximum temperature, annual, minimum and maximum precipitation, and the degree of seasonality in temperature and precipitation (defined in Supporting Information Table S5). Climate data were aggregated for each species by first retaining unique localities only to avoid pseudo‐replication, and second, by averaging the climate data across these unique localities to obtain species means (Supporting Information Table S6). Species' altitudinal median and range were derived from field observations reported in the literature (data and sources are in Supporting Information Table S7).

Macaques can broadly be divided into two ecological groups: species that predominantly occur in broadleaf evergreen (BE) forests, and those that exist in a wide range of forest and nonforest (non‐BE) habitats (Fooden, 1982). In addition, habitat breadth, dietary breadth, the degree of frugivory and folivory, and the range in the proportion of fruits in the diet were collated from the literature. Finally, mean male and female adult body masses were retrieved to have a measure of overall body size. Data (including sources) on habitat, diet, and body mass are presented in Supporting Information Tables S8, S9, and S10, respectively.

#### 2.1.2 Within‐species analysis

**Figure 1:**
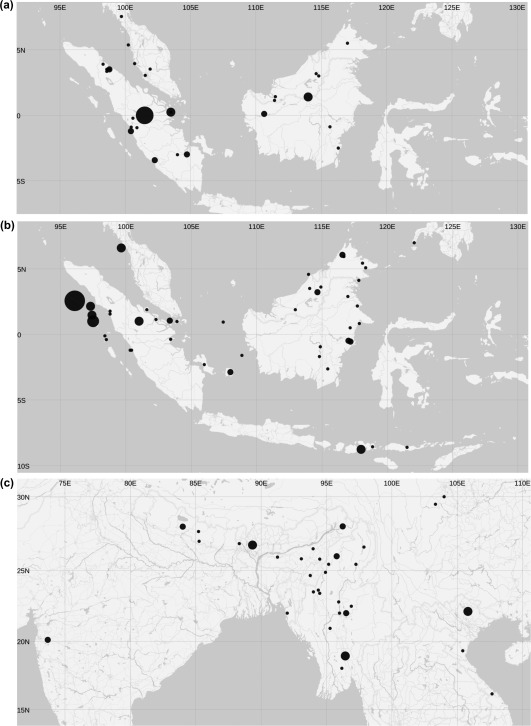
Maps showing the distribution of our sample of adult and wild‐caught specimens for (a) *M. nemestrina*, (b) *M. fascicularis*, and (c) *M. mulatta*. Bubble size is standardized across the subfigures and represents the number of specimens sampled from any one particular locality (*M. nemestrina*: *N* = 1–6; *M. fascicularis*: *N* = 1–7; *M. mulatta*: *N* = 1–3). *M. nemestrina* (28 localities) and *M. fascicularis* (45 localities) have been collected from insular Southeast Asia (Borneo and Sumatra and surrounding islands), occupying tropical habitats, and *M. mulatta* (33 localities) has been sampled from subtropical and temperate localities in India, through to Myanmar, Nepal, and Vietnam, up to China

For the intraspecific analysis we used those species for which adequate spatial and climatic variation exist in our sample. These are *Macaca nemestrina* (*N* = 43 from 28 unique localities), *M. fascicularis* (*N* = 70 from 45 unique localities) and *M. mulatta* (*N* = 44 from 33 unique localities). Only morphological data pertaining to adult, wild specimens with known provenience were used. Figure 1 depicts the geographical distribution of the sample for the three species. An interactive version of these maps showing the topography of the land surface and the sea bed can be accessed at https://nicolegrunstra.github.io/GeoMaps_3_species/. Because of damage to specimens there were missing data, which we substituted using Expectation‐Maximization (EM) imputation (Dempster et al., 1977; Gunz et al., 2009). EM imputation uses an iterative algorithm that calculates maximum‐likelihood estimates for missing values based on the covariance structure of the observed data.

Because of the paucity of data on ecological variation between populations within species, only elevation and climate were used in the intraspecific analysis. Both types of data were gathered from the WorldClim database (Hijmans et al., 2005) and analyzed on specimen level. The same eight climatic variables as in the between‐species analysis were used. Latitude and longitude were derived from the specimens' locality data.

### 2.2 Statistical analysis

Because the association between sexual dimorphism and ecogeography is outside of the scope of this article, males and females were pooled in the between‐species analysis (the species samples were approximately balanced regarding sex). For the within‐species analysis, sex differences were removed by subtracting the value of each male specimen from the male mean, and each individual female value from the female mean (by species).

In both the between‐ and the within‐species analyses, we employed two‐block partial least squares analysis (2B‐PLS; Bookstein et al., 2003; Mitteroecker and Bookstein, 2007; Rohlf and Corti, 2000) to investigate the multivariate relationship between the environment (block 1) and the morphometric data (block 2). The 2B‐PLS finds axes of successively maximum covariance between blocks. The first dimension of 2B‐PLS represents the pattern of environmental variables—determined by the environmental loadings—that has maximum covariance with the corresponding morphological pattern (determined by the morphological loadings). The individual scores along these patterns (linear combinations of the measured variables) are referred to as environmental and morphological latent variables (henceforth LVs). The axes (loading vectors) of the second dimension are orthogonal (i.e., geometrically independent) to those of the first dimension, and account for the second highest covariance between blocks, and similarly for further dimensions. The 2B‐PLS is a common approach in morphometrics and can also be applied when the number of variables exceeds sample size (as is the case in the between‐species analysis). Environmental variables include all contextual variables except for latitude and longitude, and were scaled to unit variance to eliminate differences in measurement scale. Morphological measurements were log‐transformed by the natural logarithm.

Even though 2B‐PLS has also been used to study the multivariate association of morphological and geographic variables (Frost et al., 2003), we use a different method here. In ecology, such an association is typically construed as a spatial cline or gradient, represented by the slope of the surface that results from mapping a particular biological variable on a geographic map. Locally, this slope can be estimated by regressing the variable on both latitude and longitude. The two resulting partial regression coefficients (one for latitude, one for longitude) determine the spatial direction with maximum regression slope, i.e., with the steepest local gradient on the surface. In the current multivariate context, this translates into finding a linear combination of morphological variables that has maximum slope when regressed on a linear combination of geographic coordinates. This is achieved by a singular value decomposition of the *p* × 2 matrix of partial regression coefficients of all *p* morphological variables on latitude and longitude (for a mathematical proof see Mitteroecker et al., 2016). The singular values equal the maximal slopes, and the singular vectors contain the morphological and geographic loadings that determine the corresponding LVs. This approach is similar to reduced rank regression (Izenman, 1975), hence we use this name to refer to our multivariate strategy here. Similarly to 2B‐PLS, reduced rank regression yields two pairs of latent variables in this application, but they maximize the regression slope (not the covariance) of the morphological LV on the geographic LV, and the LVs are uncorrelated, not orthogonal as in 2B‐PLS. Furthermore, in contrast to reduced rank regression, 2B‐PLS would be largely driven by the actual geographic variation (if the habitat range of a species was much wider in one direction than another, the first dimension of PLS would be aligned with this direction of maximal spatial variation, rather than the direction of the steepest cline).

Because of their phylogenetic history, species' data are not statistically independent (Felsenstein, 1985). We therefore performed a phylogenetic 2B‐PLS and reduced rank regression by a PGLS‐based algorithm (Adams and Felice, 2014; Mitteroecker et al., 2016). An independently derived molecular phylogeny of the macaque species in the sample (Arnold et al., 2010) was used for the phylogenetic correction (Supporting Information Figure S1) and aligns with other published phylogenies (Chatterjee et al., 2009; Springer et al., 2012; Tosi et al., 2003). To investigate how the effect of phylogeny on macaque morphology manifests itself in the associative patterns, we carried out the between‐species analyses first without and then with phylogenetic correction. Phylogenetic branch lengths were scaled proportional to time, assuming Brownian Motion evolution.

Lastly, in the absence of strong selection and when gene flow decreases with geographic distance, patterns of isolation by distance (IBD) emerge between populations of the same species. To test for IBD, we carried out Mantel tests on the geographic and phenotypic distance matrices within *M. nemestrina*, *M. fascicularis*, and *M. mulatta* separately, with 10,000 random permutations each. Geographic distances were represented by geodesic distances, and multivariate phenotypic distances were computed as Euclidean distances of both the original and log‐transformed measurements. All statistical analyses were carried out in Mathematica [9.0] (Wolfram Research Inc., 2012).

## 3 RESULTS

### 3.1 Between‐species analysis

#### 3.1.1 Environment

Without phylogenetic correction, 2B‐PLS yielded two latent variables that accounted for 63% (LV 1) and 34% (LV 2) of the squared covariance between the blocks of variables (see scree plot in Supporting Information Figure S2a). The correlation between blocks was strong along both dimensions, with LV 1: *r* = 0.81, and LV 2: *r* = 0.81 (we do not report *p*‐values for the interspecific analyses as their meaning is limited in this small sample of selected species). After phylogenetic correction, however, 2B‐PLS extracted only one latent variable (LV 1) with considerable covariance; in fact it accounted for 94% of the total squared covariance between blocks (*r* = 0.67). The contribution of LV 2 decreased to 4% (see scree plot in Supporting Information Figure S2b), despite a strong correlation between blocks (*r* = 0.85).

**Figure 2:**
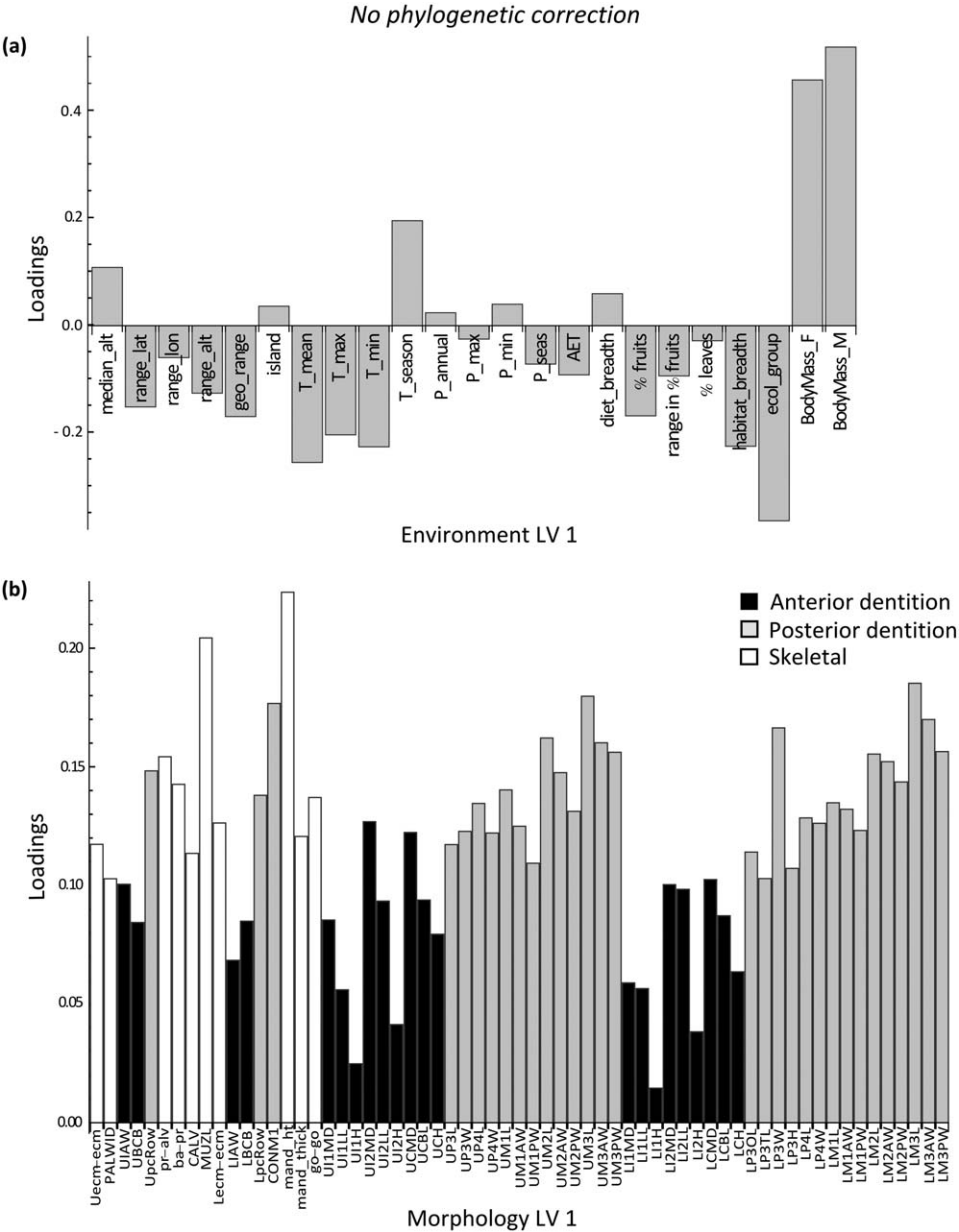
Results of the two‐block partial least squares (2B‐PLS) analysis. Latent variable (LV) 1 describes the pattern of maximum covariance between environment and morphology prior to phylogenetic correction. (**a**) Environment loadings, and (**b**) morphology loadings onto LV 1. This first factor primarily represents the association between low temperature, high temperature seasonality, and large body mass with large craniodental size (all morphological loadings are positive)

**Figure 3:**
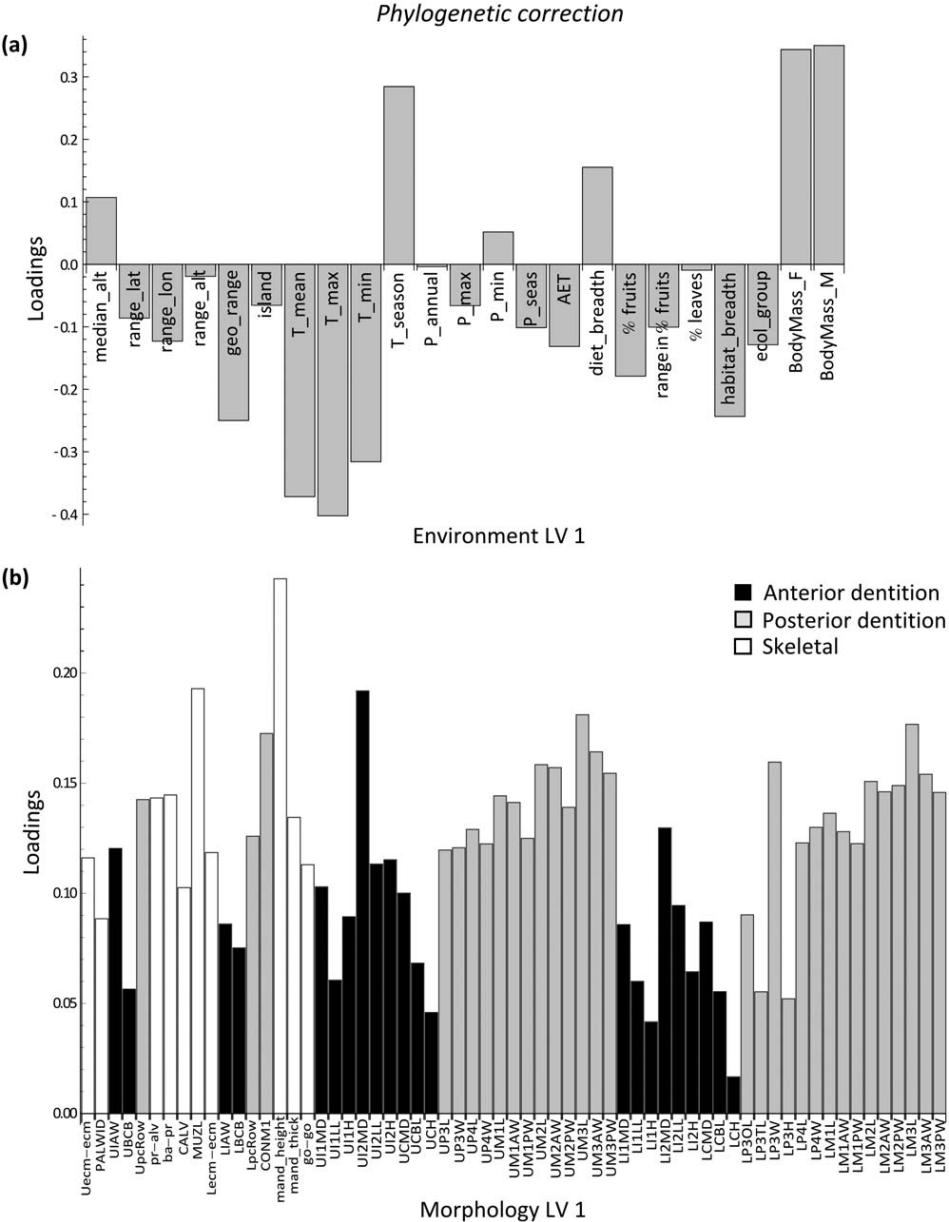
Results of the phylogenetic two‐block partial least squares (2B‐PLS) analysis. Latent variable (LV) 1 describes the pattern of maximum covariance between environment and morphology after correcting for phylogenetic relationships among the species. (**a**) Environment loadings, and (**b**) morphology loadings onto LV 1. Note the similarity in patterns with Figure 2

The PLS loadings of the environmental and morphological variables on LV 1 showed a very similar pattern before and after phylogenetic adjustment (Figures 2 and 3). Body size and temperature seasonality had high positive loadings, whereas temperature (mean, maximum, and minimum), geographic range size, habitat breadth, and ecological group had high negative loadings on LV 1. All morphological variables loaded positively on LV 1, a common allometric (i.e., overall size) effect (Mitteroecker et al., 2012). The PLS scores in Supporting Information Figure S3 further show that macaques vary along a size gradient from small‐bodied species (e.g., *M. fascicularis* and *M. sinica*) to larger‐bodied species (e.g., *M. sylvanus* and *M. fuscata*).

**Figure 4:**
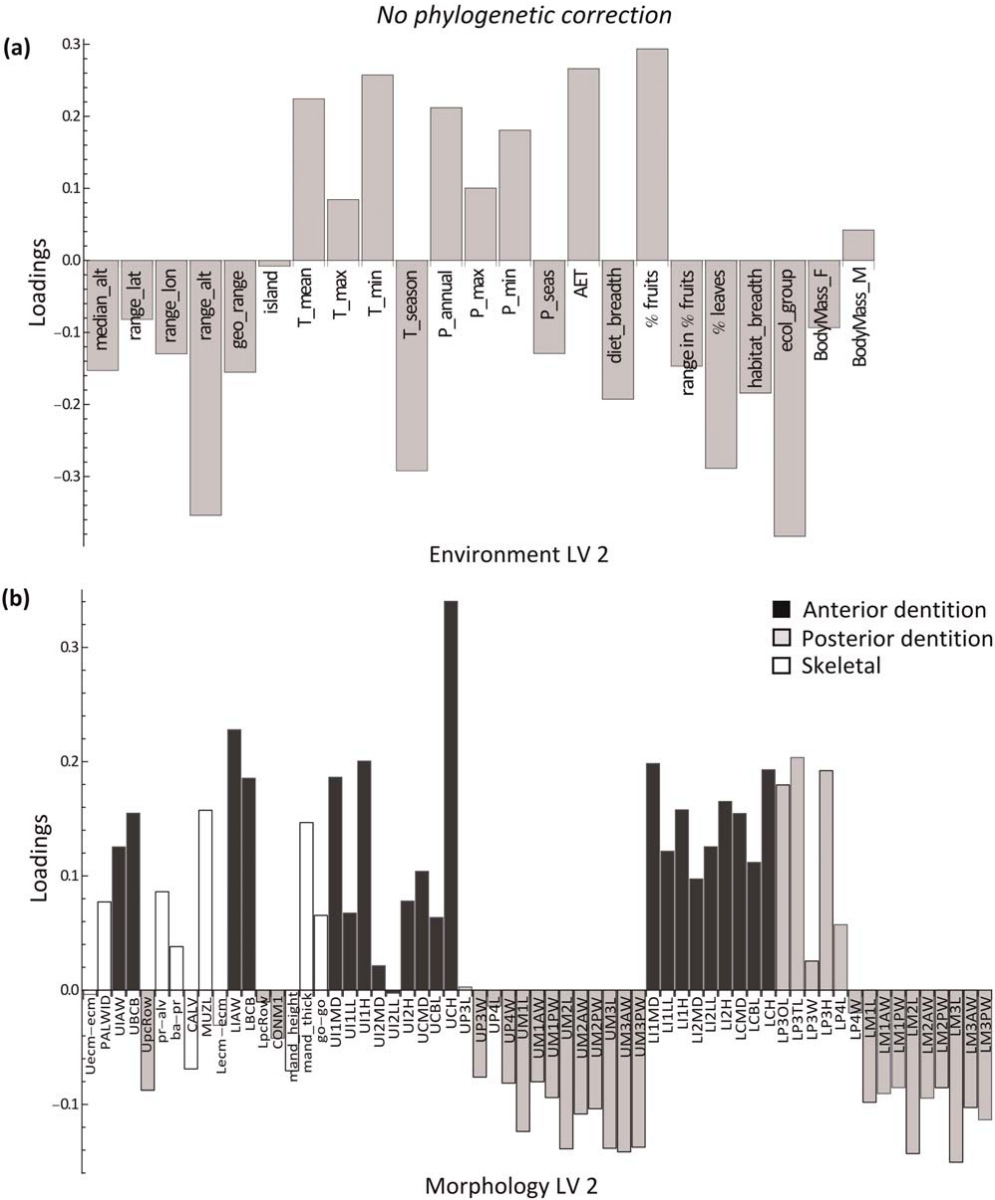
Results of the two‐block partial least squares (2B‐PLS) analysis. Latent variable (LV) 2 describes the next largest covariance between environment and morphology prior to phylogenetic correction. (**a**) Environment loadings, and (**b**) morphology loadings onto LV 2. This second, primarily ecological factor represents the association of high rainfall and habitat productivity, low rainfall seasonality, a high percentage of fruits in the diet, low variation in the amount of fruits, and a low percentage of leaves in the diet with an antero‐posterior dental contrast

**Figure 5:**
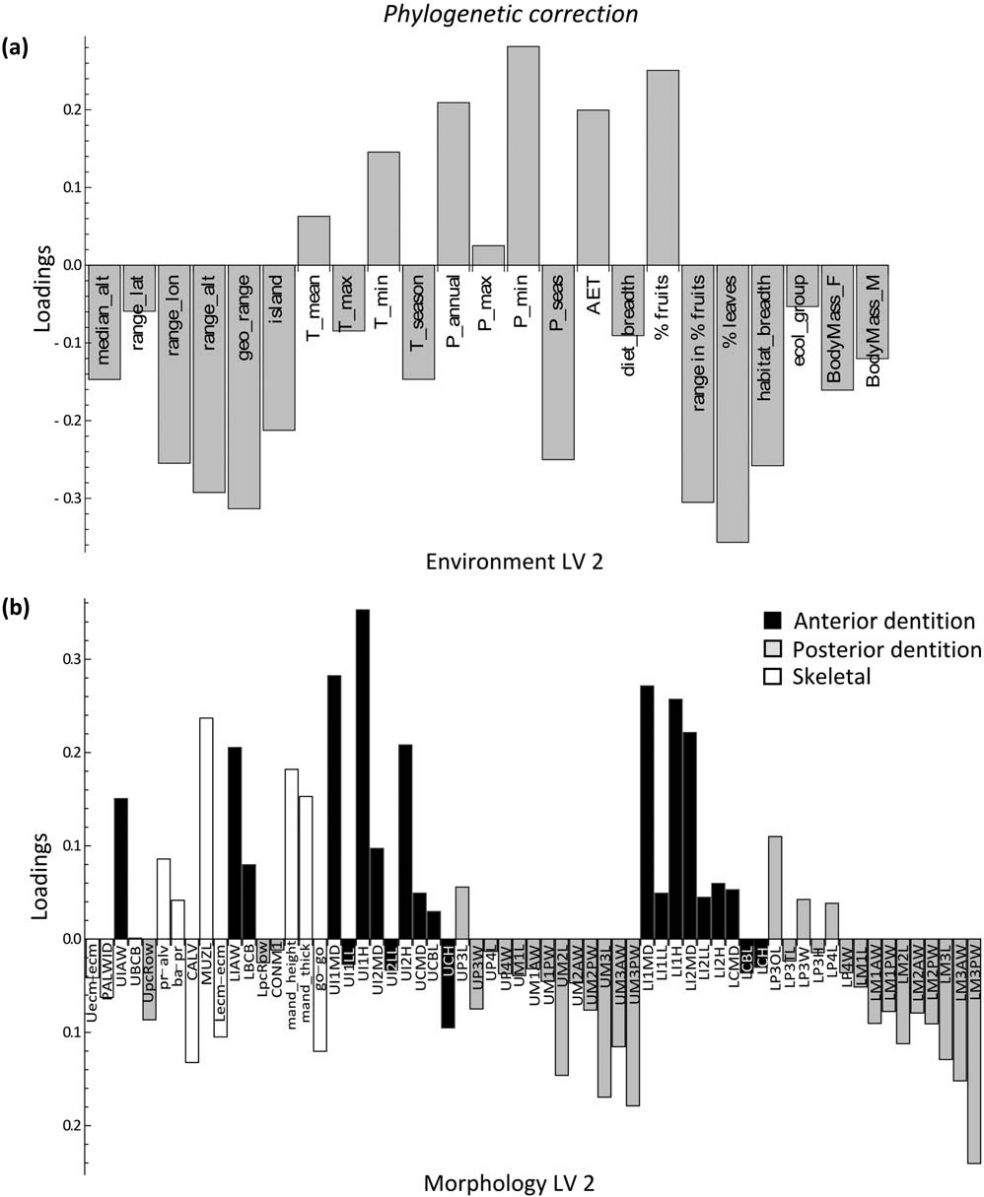
Results of the phylogenetic two‐block partial least squares (2B‐PLS) analysis. Latent variable (LV) 2 describes the next largest covariance between environment and morphology after phylogenetic correction. (**a**) Environment loadings, and (**b**) morphology loadings onto LV 2. Despite the similarity of the patterns to those in Figure 4, LV 2 is diminished in effect size after phylogenetic correction (see also the scree plot in Supporting Information Figure S2)

The loading patterns of LV 2 also showed a highly similar pattern before and after accounting for phylogeny (Figures 4 and 5). Annual and minimum precipitation, minimum temperature, AET, and percentage of fruit in the diet had relatively high positive loadings for LV 2, whereas range variables, seasonality, measures of dietary variability, and percentage of leaves in the diet all loaded negatively on LV 2. The associated craniodental pattern showed a tooth size contrast (Figures 4b and 5b). Measurements pertaining to the anterior dentition loaded positively on LV 2, i.e., in the same direction as precipitation levels and percentage of fruits. Conversely, measurements of the posterior dentition loaded negatively on LV 2, in the same direction as precipitation seasonality and percentage of leaves. A larger anterior dentition is thus associated with fruit‐eating and high degree of rainfall, whereas a larger posterior dentition is associated with more leaf‐eating and drier, more seasonal environments. As mentioned, however, the association between blocks along this dimension was greatly diminished once phylogeny was taken into account.

#### 3.1.2 Geography

**Figure 6:**
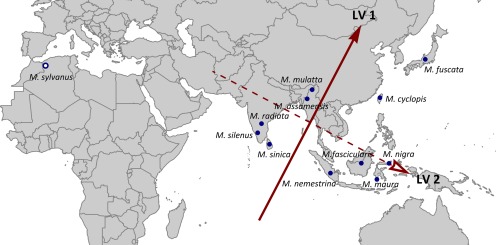
Results of the reduced rank regression after phylogenetic correction. The loading vectors prior to correction are highly similar and these results are therefore not displayed. *M. sylvanus* was omitted due to its outlying geographical position, and thus *N* = 11. Latent variable (LV) 1, the direction with the steepest morphological cline, corresponds to a south(west)‐to‐north(east) gradient, and LV 2 to a (north)west‐to‐(south)east gradient

**Figure 7:**
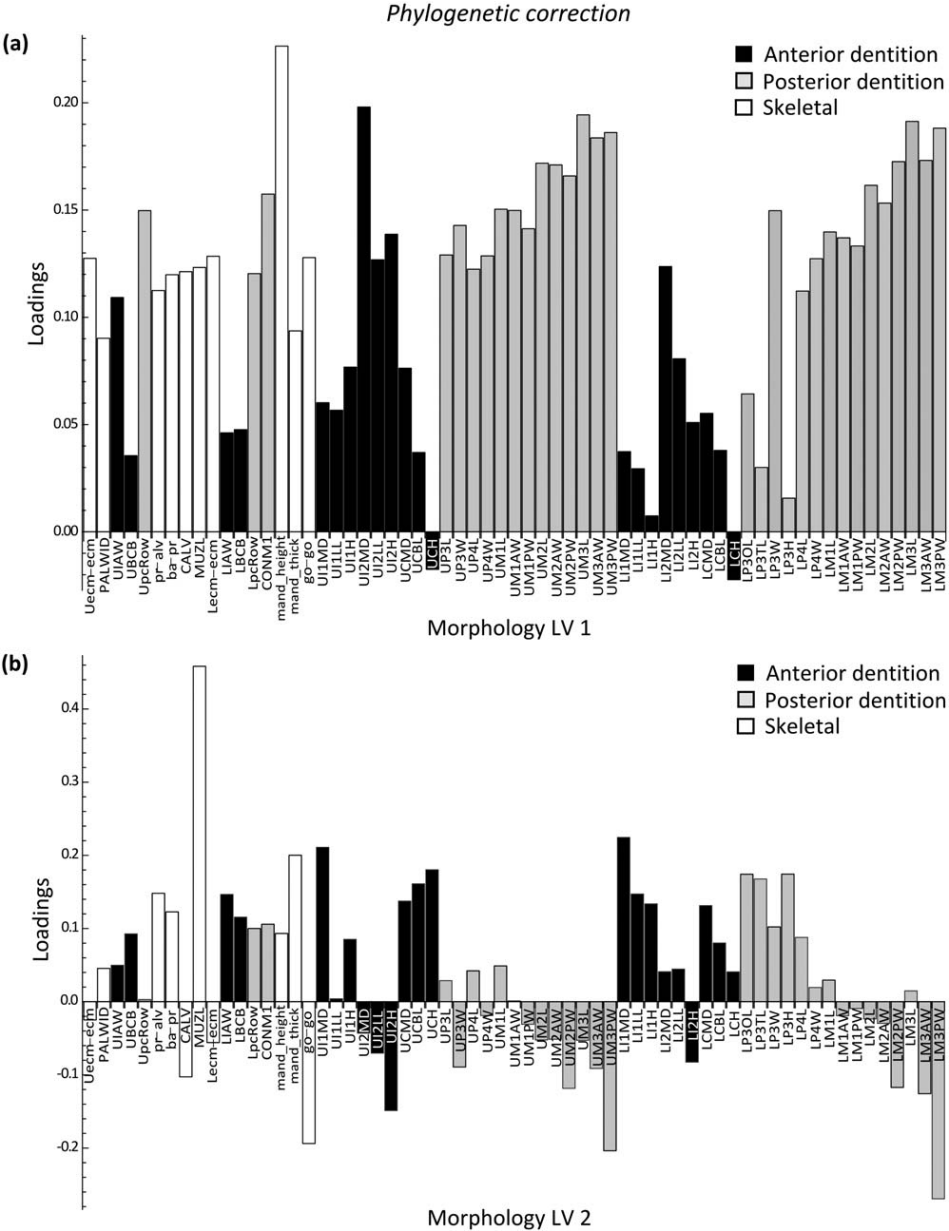
Results from the reduced rank regression with phylogenetic correction. This figure shows the loadings of the morphological variables onto latent variable (LV) 1 (**a**) and LV 2 (**b**). LV 2 is diminished in effect size after phylogenetic correction (see the scree plot in Supporting Information Figure S4), and therefore this pattern (b) warrants only limited interpretation. LV 1, on the other hand, describes the association between latitude and overall craniodental size (all morphological loadings are positive, with the exception of canine height)

The results of the reduced rank regression after phylogenetic correction are presented in Figures 6 and 7. Without phylogenetic adjustment, the results were once again highly similar and they are therefore not presented here. North African *M. sylvanus* was omitted due to its outlying geographic location. The spatial vectors representing LV 1 and LV 2 strongly corresponded to latitude and longitude, respectively (Figure 6). Prior to phylogenetic correction, LV 1 accounted for 75% of the association (total squared regression slopes) between spatial geography and morphology (*r* = 0.91) and LV 2 for the remaining 25% (*r* = 0.41) (Supporting Information Figure S4). After phylogenetic correction, however, LV 1 accounted for nearly 100% of the association, whereas LV 2 has now become negligible (see Supporting Information Figure S4). The effect of phylogeny is further shown by the drop in strength of the correlation coefficient along LV 1 (from 0.91 to 0.39).

Before as well as after phylogenetic adjustment, all craniodental measurements (with a few exceptions) were positively correlated with LV 1 (Figure 7a). Thus, macaque teeth and skulls tend to get larger along a roughly south‐to‐north gradient. LV 2 was positively associated with measurements pertaining to the anterior teeth and muzzle, and negatively with posterior tooth measurements and calvarium length (Figure 7b). LV 2 thus discriminates between a relatively larger posterior dentition in the west and a relatively larger anterior dentition in the east, although the effect size of this association is very weak when phylogeny is taken into account.

### 3.2 Within‐species analysis

#### 3.2.1 Environment

The 2B‐PLS returned no significant linear combinations between climate, altitude, and morphology for any of the three species (LV 1: *M. nemestrina: r =* 0.27*, p =* 0.40; *M. fascicularis: r =* 0.40, *p* =0.22; and *M. mulatta: r =* 0.35, *p* = 0.28). Scatter plots of the PLS scores revealed no discernible relationship between environment and morphology (not shown).

#### 3.2.2 Geography

Reduced rank regressions also did not find any significant associations between latitude, longitude and morphology for any of the three species (*M. nemestrina: r =* 0.20, *p* = 0.76; *M. fascicularis: r =* 0.33, *p* = 0.11; and *M. mulatta: r =* 0.36, *p* = 0.14). Furthermore, there were no visible trends in the regression plots (not shown). Lastly, we found no correlations between geographic proximity and morphological (dis)similarity (*M. nemestrina*: *r* = 0.07, *M. fascicularis*: *r* = 0.02, and *M. mulatta*: *r* = 0.01).

## 4 DISCUSSION

We detected signals of geography, climate, and ecology in the interspecific variation of macaque craniodental morphology, although these patterns are variably mediated by phylogeny. Our between‐species analyses demonstrated the presence of only two environmental and spatial gradients in the macaque craniodental phenotype, despite the diversity of variables in the ecogeographic and phenotypic datasets. The first factor is dominated by overall craniodental size and varies (weakly) along a latitudinal cline, with a tendency for macaques to be smaller near the equator (e.g., *M. sinica*, *M. fascicularis*, and *M. radiata*) and larger at higher latitudes (e.g., *M. assamensis* and *M. fuscata*). Concomitant with this latitudinal cline is the positive relationship of absolute craniodental size with male and female body mass, colder temperatures, and increased temperature seasonality. Taken together, these results are in agreement with a classic Bergmann effect (Millien et al., 2006), and match the positive relationship that has been found between macaque body mass and latitude (Ito et al., 2014; Harcourt and Schreier, 2009). The observed pattern along the first axis was minimally affected by phylogenetic correction: the PLS correlation was only slightly reduced from 0.81 to 0.67, and the effect size along this dimension—the percentage explained of the total squared covariance between blocks—remained large (it changed from 63% to 94%). Furthermore, the loading on LV 1 of temperature, one of the most important variables to contribute to LV 1, even increased. The pattern in macaque craniodental size thus can barely be explained by phylogenetic effects, suggesting that convergent evolution and selection played an important role. Therefore, we interpret the variation in craniodental size—along with overall body size—as an adaptive response to variation in temperature along a latitudinal gradient, and suggest here that species differentiation in *Macaca* was associated with adaptive diversification in body size.

The second factor discriminates between species with a relatively larger anterior dentition and a more prominent muzzle, and species with a relatively larger posterior dentition and longer calvaria. (We point out that the dental contrast highlighted by LV 2 represents *relative* craniodental size, because differences due to overall size are captured primarily by LV 1.) The lower third premolar (P_3_) is part of the CP_3_ honing complex in Old World monkeys (Swindler, 2002), and indeed loaded in the same direction as the canines rather than the posterior dentition. The negative association between relative anterior and relative posterior tooth size may reflect the underlying architecture of genetic independence found between incisors and postcanine teeth in baboons and mice (Hlusko et al., 2011). Regardless of whether we account for phylogeny or not, a larger anterior dentition is associated with tropical climates and increased habitat productivity, less variable habitats, small elevational, longitudinal and geographic ranges, and a high subsistence on fruits (e.g., in all representatives of the *silenus* clade, namely *M. nemestrina*, *M. silenus*, and the two Sulawesi macaques). A larger posterior dentition, by contrast, is observed in taxa occupying more temperate regions. These include macaques that experience increased seasonality, occupy a larger variety of habitats and altitudes across larger geographic ranges, and which subsist on proportionally more leaves and highly variable amounts of fruit (e.g., *M. mulatta*, *M. sylvanus*, and *M. fuscata*). This association between relative tooth size and ecogeography may be mediated by the functional link between diet (as influenced by climate and habitat, and hence indirectly also by latitude) and teeth. The association of range size variables with LV 2 reflects the tendency for less frugivorous (and more folivorous) macaque species to also occupy a wider range of habitats, at varying altitudes, and across larger geographical areas.

The environment‐craniodental contrast (LV 2) coincides with a longitudinal gradient as long as phylogeny is not accounted for. In fact, in contrast to the first factor, the second pattern can be explained almost entirely in phylogenetic terms. The environment and diet‐related variance in craniodental morphology was greatly reduced following phylogenetic correction, resulting in a negligible effect size of LV 2. This is similar to the reduction in effect sizes of the relationships between climate, diet, and macaque craniofacial shape obtained by Ito et al. (2014) after controlling for phylogeny. Although we find no evidence of environmental adaptation in LV 2 once we correct for phylogeny, the pattern itself is similar to what has been found in capuchin monkeys; namely that relative tooth size (of primarily the postcanine dentition) is bigger in species living in relatively cooler, drier, and more seasonal climates (Cáceres et al., 2014). Cáceres et al. (2014) suggested that species with larger teeth for their body size are able to process a broader range of food items. However, the authors did not employ phylogenetic comparative methods and therefore it is unknown whether the relationship between tooth size and environment in capuchins mostly reflects processes of adaptation or shared ancestry.

Habitat productivity and rainfall patterns were not associated with variation in macaque body size and craniodental size, in contrast to what has been found for baboons (Jolly, 2012), vervets (Cardini et al., 2007), and sifakas (Lehman et al., 2005). This discrepancy between macaques and their cercopithecine relatives in Africa may result from different degrees of variation in environmental factors in different geographic regions. For example, in the tropics and subtropics, patterns of rainfall are more variable than temperature and, hence, may have stronger effects on morphology (DeMenocal and Bloemendal, 1995). Also, many African monkeys may vary morphologically more with longitude than with latitude, because they have wider longitudinal distributions and are therefore subject to environmental variation mainly in that direction.

In addition to the species‐level analyses, we also investigated the presence of environmental and spatial gradients as well as isolation by distance (IBD) patterns within species to be able to infer what processes have been important in structuring intraspecific variation in macaques and whether these processes can also explain the variation between species. Intraspecific phenotypic variation that is correlated with environmental or geographic variation primarily reflects phenotypic plasticity, i.e., environmentally induced variation, or, if gene flow is low, genetic differences due to adaptation to local environments. An IBD pattern, by contrast, would result from strong genetic drift in the presence of reduced gene flow. IBD patterns can therefore reflect population history, an intraspecific equivalent to phylogenetic signal (Roseman and Auerbach, 2015). Intraspecific variation in body or skull length has previously been reported to correlate with latitude in *M. nemestrina* (Albrecht, 1980), *M. mulatta* (Fooden, 2000), and *M. fascicularis* (Fooden and Albrecht, [Link]; Schillaci et al., 2009). However, we found no relationships between craniodental variation, climate, and spatial geography within the three species in our sample, indicating low phenotypic plasticity in both absolute and relative craniodental size. These results also suggest that local environmental adaptation in the craniodental phenotype is either weak, perhaps due to relatively homogenous environments, or that gene flow is strong, e.g., due to intensive migration. The absence of detectable intraspecific plasticity supports our claim that the species differences along LV 1 are due to an evolved genetic basis.

Furthermore, we detected no evidence for IBD in craniodental size of *M. nemestrina*, *M. fascicularis*, or *M. mulatta*. The lack of an IBD pattern and spatial clines in these species is in contrast to the IBD found in recent modern humans (Betti et al., 2010) and the clinal variation observed in many African cercopithecid primates (Cardini et al., 2013; Dunn et al., 2013), respectively. This discrepancy with macaques may result from island effects in longtailed (*M. fascicularis)* and pigtailed macaques (*M. nemestrina)*, such as the sea straits that act as barriers to gene flow between populations (Abegg and Thierry, 2002). The rhesus macaque (*M. mulatta)*, on the other hand, unhindered by sea barriers and aided by their ability to move across a variety of habitats owing to their ecological flexibility, may exhibit strong male‐mediated gene flow between populations in the sampled region (Figure 1c; Fooden, 2000; Melnick, 1988; Melnick and Hoelzer, 1992; Tosi et al., 2002, 2003).

Among the two ecogeographic gradients, the second (LV 2) is of particular interest. The depicted interspecific association between craniodental variation and diet is in agreement with early comparative work that has linked large incisors (relative to postcanine teeth) to frugivory, and smaller incisors to folivory (Hylander, 1975; Robinson, 1954). Likewise, there is a well‐known and pervasive phenomenon among anthropoid primates that postcanine tooth size is often larger in folivores than in closely‐related frugivores relative to body or facial size (e.g., Vinyard and Hanna, 2005; Scott, 2011). More recently, this diet‐molar pattern has also been found in strepsirhines when adjusted for facial size (Scott, 2012). These two diet‐related patterns of relative tooth size are often explained as an adaptive response to masticatory challenges posed by the external properties of food items; large incisors are useful for the ingestion of large, husky, and fleshy fruits, whereas a large postcanine occlusal surface benefits the consumption of small and hard food items (e.g., nuts) or tough, fibrous foods (e.g., mature leaves) (Lucas, 2004; Ungar, 2011). Even though we recovered this classic association between dental dimensions and diet in our analysis, the interspecific differences along this pattern were strongly aligned with phylogenetic relatedness: when phylogeny is statistically accounted for, the pattern of association remains intact, but its magnitude diminishes. Hence, whereas the convergence of LV1 (distantly related taxa are phenotypically similar) provides good evidence for adaptive evolution, the interspecific association for LV 2 can be explained equally well by adaptation and by common ancestry. Phylogenetic patterns are not inconsistent with adaptation *per se* because adaptive evolution can drive both phenotypic and phyletic divergence, but they can also arise from neutral evolution. In the latter case, phenotypic differentiation occurs mainly by genetic drift and closely related taxa inherit their phenotypic similarity from their common ancestor. Based on the present data, an adaptive interpretation of the relationship between the relative size of the anterior and posterior dentition and diet is not sufficiently supported.

While significant relationships between diet and dental size have been recovered after phylogenetic correction on higher taxonomic levels (e.g., Scott, 2011, 2012), our results show that on lower taxonomic levels (like the genus level) shared ancestry may suffice to explain certain environment–phenotype associations. This point is particularly relevant in paleoanthropology where phylogenetic reconstruction is often complicated by the unresolved alpha taxonomy. The study of absolute and relative tooth size has been an important tool for the reconstruction of hominin diets and adaptive zones of closely related species (Kay, 1985; Organ et al., 2011; Robinson, 1954; Wood and Collard, 1999; and reviewed in Ungar, 2011). Our results offer a word of caution against adaptive interpretations of craniodental variation when the phylogeny of the studied taxa is either not known or not explicitly modeled in the analysis.

## Supporting information

Additional Supporting Information may be found online in the supporting information tab for this article.

Supporting Information 1

## References

[ajpa23439-bib-0001] Abegg, C. , & Thierry, B. (2002). Macaque evolution and dispersal in insular south‐east Asia. Biological Journal of the Linnean Society, 75, 555–576.

[ajpa23439-bib-0002] Adams, D. C. , & Felice, R. N. (2014). Assessing trait covariation and morphological integration on phylogenies using evolutionary covariance matrices. PLoS ONE, 9, e94335. 10.1371/journal.pone.0094335 PMC3984176 24728003

[ajpa23439-bib-0003] Albrecht, G. H. (1980). Latitudinal, taxonomic, sexual, and insular determinants of size variation in pigtail macaques, *Macaca nemestrina* . International Journal of Primatology, 1, 141–152.

[ajpa23439-bib-0004] Arnold, C. , Matthews, L. J. , & Nunn, C. L. (2010). The 10kTrees website: A new online resource for primate phylogeny. Evolutionary Anthropology, 19, 114–118.

[ajpa23439-bib-0005] Ashton, K. G. , Tracy, M. C. , & de Queroz, A. (2000). Is Bergmann's rule valid for mammals? The American Naturalist, 156, 390–415. 10.1086/303400 29592141

[ajpa23439-bib-0006] Bergmann, C. (1847). Über die Verhältnisse der Wärmeökonomie der Thiere zu ihrer Größe. Göttinger Studien, 3, 595–708.

[ajpa23439-bib-0007] Betti, L. , Balloux, F. , Hanihara, T. , & Manica, A. (2010). The relative role of drift and selection in shaping the human skull. American Journal of Physical Anthropology, 141, 76–82. 19582777 10.1002/ajpa.21115

[ajpa23439-bib-0008] Bookstein, F. L. , Gunz, P. , Mitteroecker, P. , Prossinger, H. , Schaefer, K. , & Seidler, H. (2003). Cranial integration in Homo: Singular warps analysis of the midsagittal plane in ontogeny and evolution. Journal of Human Evolution, 44, 167–187. 12662941 10.1016/s0047-2484(02)00201-4

[ajpa23439-bib-0009] Brandon‐Jones, D. (1996). The Asian Colobinae (Mammalia: Cercopithecidae) as indicators of Quaternary climatic change. Biological Journal of the Linnean Society, 59, 327–350.

[ajpa23439-bib-0010] Cáceres, N. , Meloro, C. , Carotenuto, F. , Passaro, F. , Sponchiado, J. , Melo, G. L. , & Raia, P. (2014). Ecogeographical variation in skull shape of capuchin monkeys. Journal of Biogeography, 41, 501–512.

[ajpa23439-bib-0011] Cardini, A. , Diniz Filho, J. A. F. , Polly, P. D. , & Elton, S. (2010). Biogeographic analysis using geometric morphometrics: Clines in skull size and shape in a widespread African arboreal monkey In Elewa A. (Ed.), Morphometrics for nonmorphometricians (pp. 191–217). Berlin: Springer‐Verlag.

[ajpa23439-bib-0012] Cardini, A. , Dunn, J. , O'higgins, P. , & Elton, S. (2013). Clines in Africa: Does size vary in the same way among widespread sub‐Saharan monkeys? Journal of Biogeography, 40, 370–381.

[ajpa23439-bib-0013] Cardini, A. , & Elton, S. (2009). Geographical and taxonomic influences on cranial variation in red colobus monkeys (Primates, Colobinae): Introducing a new approach to “morph” monkeys. Global Ecology and Biogeography, 18, 248–263.

[ajpa23439-bib-0014] Cardini, A. , Jansson, A.‐U. , & Elton, S. (2007). A geometric morphometric approach to the study of ecogeographical and clinal variation in vervet monkeys. Journal of Biogeography, 34, 1663–1678.

[ajpa23439-bib-0015] Chatterjee, H. J. , Ho, S. Y. W. , Barnes, I. , & Groves, C. (2009). Estimating the phylogeny and divergence times of primates using a supermatrix approach. BMC Evolutionary Biology, 9, 259. 19860891 10.1186/1471-2148-9-259 PMC2774700

[ajpa23439-bib-0016] Delson, E. (1980). Fossil macaques, phyletic relationships and a scenario of deployment In Lindburg D. G. (Ed.), The macaques: Studies in ecology, behavior and evolution (pp. 10–30). New York: Van Nostrand Reinhold.

[ajpa23439-bib-0017] DeMenocal, P. B. , & Bloemendal, J. (1995). Plio‐Pleistocene climatic variability in subtropical Africa and the palaeoenvironment of hominid evolution: A combined data‐model approach In Vrba E. S., Denton G. H., Partridge T. C., & Burckle L. H. (Eds.), Paleoclimate and evolution, with emphasis on human origins (pp. 262–288). New Haven, CT: Yale University Press.

[ajpa23439-bib-0018] Dempster, A. P. , Laird, N. M. , & Rubin, D. B. (1977). Maximum likelihood from incomplete data via the EM algorithm. Journal of the Royal Statistical Society. Series B (Methodological) Journal of the Royal Statistical Society. Series B, 39, 1–38.

[ajpa23439-bib-0019] Dunn, J. , Cardini, A. , & Elton, S. (2013). Biogeographic variation in the baboon: Dissecting the cline. Journal of Anatomy, 223, 337–352. 24028342 10.1111/joa.12085 PMC3791127

[ajpa23439-bib-0020] Felsenstein, J. (1985). Phylogenies and the comparative method. The American Naturalist, 125, 1–15. 10.1086/703055 31094602

[ajpa23439-bib-0021] Fooden, J. (1982). Ecogeographic segregation of macaque species. Primates, 23, 574–579.

[ajpa23439-bib-1022] Fooden, J. , & Albrecht G. H. (1993). Latitudinal and insular variation of skull size in crab‐eating macaques (Primates, Cercopithecidae: *Macaca fascicularis*). American Journal of Physical Anthropology, 92, 521–538. 8296879 10.1002/ajpa.1330920409

[ajpa23439-bib-0022] Fooden, J. (2000). Systematic review of the rhesus macaque, *Macaca mulatta* (Zimmermann, 1780). Fieldiana Zoology, 69, 1–180.

[ajpa23439-bib-0023] Frost, S. R. , Marcus, L. F. , Bookstein, F. L. , Reddy, D. P. , & Delson, E. (2003). Cranial allometry, phylogeography, and systematics of large‐bodied papionins (primates: Cercopithecinae) inferred from geometric morphometric analysis of landmark data. The Anatomical Record, 275A, 1048–1072. 10.1002/ar.a.10112 14613306

[ajpa23439-bib-0024] Gunz, P. , Mitteroecker, P. , Neubauer, S. , Weber, G. W. , & Bookstein, F. L. (2009). Principles for the virtual reconstruction of hominin crania. Journal of Human Evolution, 57, 48–62. 19482335 10.1016/j.jhevol.2009.04.004

[ajpa23439-bib-0025] Harcourt, A. H. , & Schreier, B. M. (2009). Diversity, body mass, and latitudinal gradients in primates. International Journal of Primatology, 30, 283–300.

[ajpa23439-bib-0026] Harvati, K. , & Weaver, T. D. (2006). Human cranial anatomy and the differential preservation of population history and climate signatures. The Anatomical Record Part A: Discoveries in Molecular, Cellular, and Evolutionary Biology, 288A, 1225–1233. 10.1002/ar.a.20395 17075844

[ajpa23439-bib-0027] Hijmans, R. J. , Cameron, S. E. , Parra, J. L. , Jones, P. G. , & Jarvis, A. (2005). Very high resolution interpolated climate surfaces for global land areas. International Journal of Climatology, 25, 1965–1978.

[ajpa23439-bib-0028] Hlusko, L. J. , Sage, R. D. , & Mahaney, M. C. (2011). Modularity in the mammalian dentition: Mice and monkeys share a common dental genetic architecture. Journal of Experimental Zoology, 316, 21–49. 20922775 10.1002/jez.b.21378 PMC3095220

[ajpa23439-bib-0029] Hylander, W. L. (1975). Incisor size and diet in anthropoids with special reference to cercopithecidae. Science, 189, 1096–1097. 10.1126/science.808855 808855

[ajpa23439-bib-0030] Ito, T. , Nishimura, T. , & Takai, M. (2014). Ecogeographical and phylogenetic effects on craniofacial variation in macaques. American Journal of Physical Anthropology, 154, 27–41. 24449333 10.1002/ajpa.22469

[ajpa23439-bib-0031] Izenman, A. J. (1975). Reduced‐rank regression for the multivariate linear model. Journal of Multivariate Analysis, 5, 248–264.

[ajpa23439-bib-0032] Jolly, C. J. (2012). Rainfall is not a genus‐wide predictor of mean body mass in baboon populations. Journal of Zoology, 286, 185–193.

[ajpa23439-bib-0033] Jones, K. E. , Bielby, J. , Cardillo, M. , Fritz, S. A. , O'dell, J. , Orme, C. D. L. , … Purvis, A. (2009). PanTHERIA: A species‐level database of life history, ecology, and geography of extant and recently extinct mammals. Ecology, 90, 2648–2648.

[ajpa23439-bib-0034] Kamilar, J. M. , Muldoon, K. M. , Lehman, S. M. , & Herrera, J. P. (2012). Testing Bergmann's rule and the resource seasonality hypothesis in malagasy primates using GIS‐based climate data. American Journal of Physical Anthropology, 147, 401–408. 22271559 10.1002/ajpa.22002

[ajpa23439-bib-0035] Kay, R. F. (1985). Dental evidence for the diet of Australopithecus. Annual Review of Anthropology, 14, 315–341.

[ajpa23439-bib-0036] Lehman, S. M. , Mayor, M. , & Wright, P. C. (2005). Ecogeographic size variations in Sifakas: A test of the resource seasonality and resource quality hypotheses. American Journal of Physical Anthropology, 126, 318–328. 15386235 10.1002/ajpa.10428

[ajpa23439-bib-0037] Lucas, P. W. (2004). Dental functional morphology: How teeth work. Cambridge: Cambridge University Press.

[ajpa23439-bib-0038] Mayr, E. (1956). Geographical character gradients and climatic adaptation. Evolution, 10, 105–108.

[ajpa23439-bib-0039] Meiri, S. (2011). Bergmann's rule—What's in a name? Global Ecology and Biogeography, 20, 203–207.

[ajpa23439-bib-0040] Meiri, S. , & Dayan, T. (2003). On the validity of Bergmann's rule. Journal of Biogeography, 30, 331–351.

[ajpa23439-bib-0041] Melnick, D. J. (1988). The genetic structure of a primate species: Rhesus macaques and other Cercopithecine monkeys. International Journal of Primatology, 9, 195–231.

[ajpa23439-bib-0042] Melnick, D. J. , & Hoelzer, G. A. (1992). Differences in male and female macaque dispersal lead to contrasting distributions of nuclear and mitochondrial DNA variation. International Journal of Primatology, 13, 379–393.

[ajpa23439-bib-0043] Meloro, C. , Cáceres, N. , Carotenuto, F. , Sponchiado, J. , Melo, G. L. , Passaro, F. , & Raia, P. (2014). In and out the Amazonia: Evolutionary ecomorphology in howler and capuchin monkeys. Evolutionary Biology, 41, 38–51.

[ajpa23439-bib-0044] Millien, V. , Lyons, K. , Olson, L. , Smith, F. A. , Wilson, A. B. , & Yom‐Tov, Y. (2006). Ecotypic variation in the context of global climate change: Revisiting the rules. Ecology Letters, 9, 853–869. 16796576 10.1111/j.1461-0248.2006.00928.x

[ajpa23439-bib-0045] Mitteroecker, P. , & Bookstein, F. (2007). The conceptual and statistical relationship between modularity and morphological integration. Systematic Biology, 56, 818–836. 17934997 10.1080/10635150701648029

[ajpa23439-bib-0046] Mitteroecker, P. , Cheverud, J. M. , & Pavlicev, M. (2016). Multivariate analysis of genotype–phenotype association. Genetics, 202, 1345–1363. 26896328 10.1534/genetics.115.181339 PMC4905550

[ajpa23439-bib-0047] Mitteroecker, P. , Gunz, P. , Neubauer, S. , & Müller, G. B. (2012). How to explore morphological integration in human evolution and development? Evolutionary Biology, 39, 536–553.

[ajpa23439-bib-0048] Organ, C. , Nunn, C. L. , Machanda, Z. , & Wrangham, R. W. (2011). Phylogenetic rate shifts in feeding time during the evolution of Homo. Proceedings of the National Academy of Sciences of the United States of America, 108, 14555–14559. 21873223 10.1073/pnas.1107806108 PMC3167533

[ajpa23439-bib-0049] Rensch, B. (1938). Some problems of geographical variation and species‐formation. Proceedings of the Linnean Society of London, 150, 275–285.

[ajpa23439-bib-0050] Robinson, J. T. (1954). Prehominid dentition and hominid evolution. Evolution, 8, 324–334.

[ajpa23439-bib-0051] Rohlf, F. J. , & Corti, M. (2000). Use of two‐block partial least‐squares to study covariation in shape. Systematic Biology, 49, 740–753. 12116437 10.1080/106351500750049806

[ajpa23439-bib-0052] Roseman, C. C. , & Auerbach, B. M. (2015). Ecogeography, genetics, and the evolution of human body form. Journal of Human Evolution, 78, 80–90. 25456824 10.1016/j.jhevol.2014.07.006

[ajpa23439-bib-0053] Schillaci, M. A. , Meijaard, E. , & Clark, T. (2009). The effect of island area on body size in a primate species from the Sunda Shelf Islands. Journal of Biogeography, 36, 362–371.

[ajpa23439-bib-0054] Scott, J. E. (2011). Folivory, frugivory, and postcanine size in the cercopithecoidea revisited. American Journal of Physical Anthropology, 146, 20–27. 21710656 10.1002/ajpa.21535

[ajpa23439-bib-0055] Scott, J. E. (2012). Molar size and diet in the Strepsirrhini: Implications for size‐adjustment in studies of primate dental adaptation. Journal of Human Evolution, 63, 796–804. 23098627 10.1016/j.jhevol.2012.09.001

[ajpa23439-bib-0056] Springer, M. S. , Meredith, R. W. , Gatesy, J. , Emerling, C. A. , Park, J. , Rabosky, D. L. , … Murphy, W. J. (2012). Macroevolutionary dynamics and historical biogeography of primate diversification inferred from a species supermatrix. PloS One, 7, e49521. 23166696 10.1371/journal.pone.0049521 PMC3500307

[ajpa23439-bib-0057] Swindler, D. R. (2002). Primate dentition: An introduction to the teeth of non‐human primates. Cambridge: Cambridge University Press.

[ajpa23439-bib-0058] Tosi, A. J. , Morales, J. C. , & Melnick, D. J. (2002). Y‐chromosome and mitochondrial markers in *Macaca fascicularis* indicate introgression with Indochinese *M. mulatta* and a biogeographic barrier in the Isthmus of Kra. International Journal of Primatology, 23, 161–178.

[ajpa23439-bib-0059] Tosi, A. J. , Morales, J. C. , & Melnick, D. J. (2003). Paternal, maternal, and biparental molecular markers provide unique windows onto the evolutionary history of macaque monkeys. Evolution, 57, 1419–1435. 12894949 10.1111/j.0014-3820.2003.tb00349.x

[ajpa23439-bib-0060] Ungar, P. S. (2011). Dental evidence for the diets of plio‐pleistocene hominins. Yearbook of Physical Anthropology, 54, 47–62. 10.1002/ajpa.21610 22101687

[ajpa23439-bib-0061] Viguier, B. (2004). Functional adaptations in the craniofacial morphology of Malagasy primates: Shape variations associated with gummivory in the family Cheirogaleidae. Annals of Anatomy, 186, 495–501. 15646283 10.1016/S0940-9602(04)80093-1

[ajpa23439-bib-0062] Vinyard, C. J. , & Hanna, J. (2005). Molar scaling in strepsirrhine primates. Journal of Human Evolution, 49, 241–269. 15935438 10.1016/j.jhevol.2005.04.002

[ajpa23439-bib-0063] Wolfram Research Inc . (2012). Mathematica. Champaign (IL): Wolfram Research, Inc.

[ajpa23439-bib-0064] Wood, B. , & Collard, M. (1999). The human genus. Science, 284, 65–71. 10102822 10.1126/science.284.5411.65

