# Supplementary material for: A multivariate ecogeographic analysis of macaque craniodental variation
Source: Am J Phys Anthropol. 2018 Feb 15;166(2):386–400. doi: 10.1002/ajpa.23439 (PMC6492120; doi:10.1002/ajpa.23439)
Supplement: Supplementary file 1 — Supporting Information 1 [file AJPA-166-386-s001.docx]

*American Journal of Physical Anthropology*

**SUPPORTING INFORMATION**

**A Multivariate Ecogeographic Analysis of Macaque Craniodental Variation**

Nicole D.S. Grunstra, Philipp Mitteroecker, Robert A. Foley

**Table S1** Definition of mesiodistal tooth length measurements.

| **Tooth class** |  | **Measurement description** |
| --- | --- | --- |
| Incisors |  | From most mesial to most distal corner along incisal edge. |
| Canines | *Upper* | From most mesial point on mesial edge (at the cemento-enamel junction (CEJ)) to distolingual border/corner, parallel to the jaw. |
|  | *Lower* | From mesial to distolabial surface at level of mesial alveolar margin. |
| Premolars |  | From mesial to distal border (or contact point). |
|  | *P_3_* (2x) | (1) occlusal length, from mesio-occlusal corner to distal contact point; (2) total length, from mesiobuccal CEJ to distal contact point on occlusal surface. |
| Molars |  | From mesial to distal border (or contact point), along the mesiodistal mid-line of the crown. |

N.B. All measurements were taken irrespective of tooth orientation in the jaw.

**Table S2** Definition of buccolingual tooth width measurements.

| **Tooth class** |  | **Measurement description** |
| --- | --- | --- |
| **Incisors** |  | From lingual cemento-enamel junction (CEJ) to labial CEJ. |
| **Canines** |  | As per incisors; maximum width. |
| **Premolars** |  | From most convex point on lingual to most convex point on buccal surface, measured mid-crown. |
|  | *P_3_* | As per upper, but at distal (non-honing face) part of crown. |
| **Molars** |  | From most convex point on lingual to most convex point on buccal surface, measured mid- crown (2x), at (1) mesial and (2) distal cusps. |

N.B. All measurements captured maximum width, typically at the most bulbous part of the crown, and were taken at a right angle of the tooth length measurement (except in the case of the incisors).

**Table S3** Definition of crown height measurements.

| **Tooth (class)** | **Measurement description** |
| --- | --- |
| Incisors | From cemento-enamel junction (CEJ) to occlusal edge. |
| Canines | From CEJ on mesiolabial edge/groove to cusp apex. |
| *P_3_* | From mesiobuccal CEJ to cusp apex. |

**Table S4** Coordinates of the central point in each species' geographic range ('mid lat' and 'mid lon'; in decimal degrees), the ranges (in decimal degrees) in latitude and longitude occupied, the total geographic range size, mean actual evapotranspiration rate (AET), and island occurrence of each macaque species used in this study. All data from the panTHERIA database (Jones et al., 2009), except for island occurrence, which was coded by NDSG based on known species distributions.

| **species** | **mid lat (DD)** | **mid lon (DD)** | **lat range (DD)** | **lon range (DD)** | **geo range (km^2^)** | **AET (mm)** | **island*^1^*** |
| --- | --- | --- | --- | --- | --- | --- | --- |
| *M. assamensis* | 23.01 | 97.19 | 16.15 | 27.35 | 1383530 | 1021 | 3 |
| *M. cyclopis* | 23.91 | 121.04 | 1.90 | 0.82 | 12815 | 800 | 1 |
| *M. fascicularis* | 4.13 | 111.24 | 29.02 | 32.12 | 2197587 | 1553 | 2 |
| *M. fuscata* | 35.88 | 135.51 | 11.32 | 11.92 | 177386 | 793 | 1 |
| *M. maura* | -4.98 | 119.90 | 1.45 | 1.11 | 13649 | 1200 | 1 |
| *M. mulatta* | 25.07 | 96.67 | 22.76 | 50.92 | 6566547 | 902 | 3 |
| *M. nemestrina* | 3.04 | 108.07 | 15.87 | 22.39 | 1033483 | 1681 | 2 |
| *M. nigra* | 1.01 | 124.26 | 1.48 | 1.96 | 13015 | 1600 | 1 |
| *M. radiata* | 14.59 | 77.06 | 13.06 | 8.82 | 679816 | 845 | 3 |
| *M. silenus* | 13.19 | 76.12 | 6.50 | 3.00 | 56995 | 953 | 3 |
| *M. sinica* | 7.87 | 80.78 | 3.60 | 2.00 | 66626 | 1537 | 1 |
| *M. sylvanus* | 34.21 | -0.62 | 5.50 | 13.33 | 95331 | 453 | 3 |

***^1^*** 1 = occurs on island(s) only, 2 = mixed occurrence (island and continental), 3 = occurs on continental mainland only.
*M. mulatta* (the rhesus macaque) occurs on a small shallow-water island (Hainan) close to the Chinese mainland, which it likely reached in the last few centuries since human occupation, as well as on Cayo Santiago off the coast of Puerto Rico, where it is was introduced. Nevertheless, for our purposes, the rhesus macaque qualifies as a continental mainland species.

**Table S5** Climate variables used in this study (extracted from the WorldClim database; Hijmans et al., 2005).

| **Variable** | **WorldClim** | **Definition** | **units** |
| --- | --- | --- | --- |
| T mean | BIO 1 | Annual mean temperature | °C * 10 |
| T max | BIO 5 | Maximum temperature of warmest month | °C * 10 |
| T min | BIO 6 | Minimum temperature of coldest month | °C * 10 |
| T seasonal | BIO 4 | Temperature seasonality (standard deviation) | (SD * 100) |
| P annual | BIO 12 | Annual precipitation | mm |
| P max | BIO 13 | Precipitation of wettest month | mm |
| P min | BIO 14 | Precipitation of driest month | mm |
| P seasonal | BIO 15 | Precipitation seasonality (coefficient of variation) | (CV) |

**Table S6** Climate data on temperature, precipitation, and seasonality for the macaque species used in this study. Data were derived from the WorldClim database (Hijmans et al. 2005) on the basis of sampled localities of specimens of wild (not captive) origin in our sample. See Materials and Methods (main paper) and Table S4 for further details.

| **species** | **T mean** | **T max** | **T min** | **T seas** | **P annual** | **P max** | **P min** | **P seas** |
| --- | --- | --- | --- | --- | --- | --- | --- | --- |
| *M. assamensis* | 177 | 264 | 49 | 4149 | 2260 | 538 | 8 | 95 |
| *M. cyclopis* | 240 | 302 | 162 | 2619 | 2717 | 594 | 46 | 89 |
| *M. fascicularis* | 259 | 308 | 212 | 356 | 2742 | 331 | 141 | 29 |
| *M. fuscata* | 106 | 256 | -42 | 7663 | 2234 | 336 | 77 | 46 |
| *M. maura* | 253 | 309 | 195 | 376 | 2565 | 447 | 53 | 61 |
| *M. mulatta* | 226 | 322 | 96 | 3986 | 1933 | 438 | 9 | 93 |
| *M. nemestrina* | 260 | 311 | 211 | 325 | 2888 | 343 | 145 | 27 |
| *M. nigra* | 258 | 310 | 212 | 338 | 2710 | 368 | 119 | 33 |
| *M. radiata* | 231 | 312 | 152 | 1670 | 1542 | 372 | 12 | 79 |
| *M. silenus* | 245 | 320 | 178 | 1286 | 2589 | 784 | 11 | 101 |
| *M. sinica* | 256 | 316 | 201 | 921 | 2047 | 339 | 56 | 55 |
| *M. sylvanus* | 147 | 323 | 13 | 6124 | 708 | 108 | 5 | 59 |

**
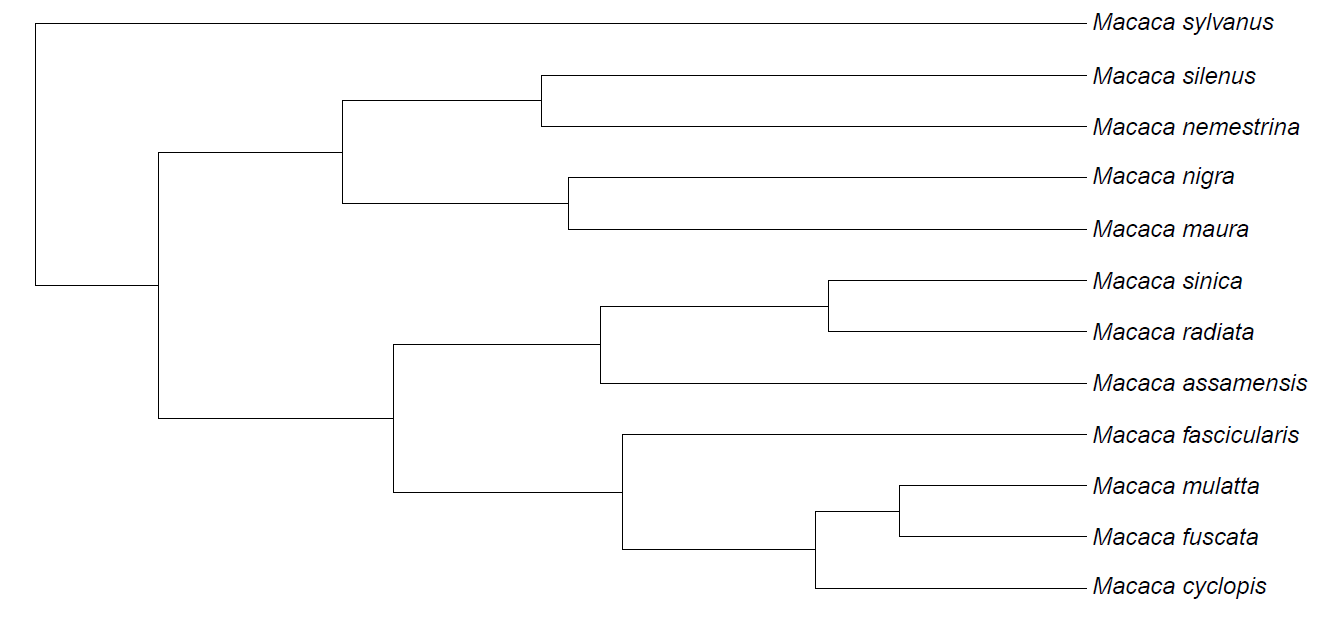

Figure S1** Molecular macaque phylogeny from the *10kTrees Project* (version 2; Arnold et al., 2010). Species not included in the study are not displayed.

**Table S7** Elevational distribution of macaques.

| **Species** | **Median altitude *^1^*** | **Altitudinal range** |  | **Sources *^2^*** |
| --- | --- | --- | --- | --- |
| *M. assamensis* | 1200 | 2750 |  | a, b, c |
| *M. cyclopis* | 1250 | 3300 |  | d, e |
| *M. fascicularis* | 200 | 2000 |  | e, f |
| *M. fuscata* | 600 | 3180 |  | e, g, h |
| *M. maura* | 250 | 2000 |  | i |
| *M. mulatta* | 500 | 4000 |  | e, j |
| *M. nemestrina* | 300 | 1700 |  | k |
| *M. nigra* | 400 | 2000 |  | i |
| *M. radiata* | 1250 | 2600 |  | b, c, l, m |
| *M. silenus* | 900 | 1200 |  | k |
| *M. sinica* | 500 | 2300 |  | b, c, n |
| *M. sylvanus* | 1900 | 2200 |  | o |

We have chosen to use species-level data as they are known from the literature, rather than values derived from our sample, because the goal of this work is to analyse the association between environment and phenotype in evolved species differences, which all members of the same species are assumed to share; the spatial distribution of the sample may not be representative of the species.
*^1^* Often the median altitude was computed based on altitudes reported for individual localities, or estimated based on locality data in combination with www.elevationmap.net.
*^2^* Data were collated from species accounts in the online database All The World's Primates (www.alltheworldprimates.org, 2011) and the following additional publications: a) Fooden (1982b), b) Fooden (1986), c) Fooden (1988), d) Fooden & Wu (2001), e) Fooden (2006), f) Fooden,(1996), g) Fooden & Aimi (2005), h) Muroyama & Yamada (2010), i) Fooden (1969), j) Fooden (2000), k) Fooden (1975), l) Fooden (1981), m) Molur et al. (2003), n) Fooden (1979), o) Fooden (2007).

**Table S8** Habitat ecology of macaques.

| **Species** | **Habitat breadth**  **(no. of layers) *^1^*** | **Ecological**  **group *^2^*** | **Sources** |
| --- | --- | --- | --- |
| *M. assamensis* | 2 | 1 | a |
| *M. cyclopis* | 3 | 2 | d, e |
| *M. fascicularis* | 7 | 2 | e, f |
| *M. fuscata* | 3 | 2 | e, g |
| *M. maura* | 3 | 1 | i |
| *M. mulatta* | 12 | 2 | e, j |
| *M. nemestrina* | 5 | 1 | k |
| *M. nigra* | 2 | 1 | i |
| *M. radiata* | 8 | 2 | b, c, l |
| *M. silenus* | 2 | 1 | k |
| *M. sinica* | 3 | 2 | b, c, n |
| *M. sylvanus* | 6 | 2 | o |

*^1^* Measured as the number of biomes that are of major importance to a species; artificial (i.e. human-made) or otherwise heavily human-impacted habitats are together only counted once. Definition of habitat layers following the IUCN Habitats Classification Scheme (IUCN Red List, 2007). Data were collated from species accounts in the IUCN Red List (www.iucnredlist.org), All The World Primates, and additional publications (*sources*; key to references can be found in Table S5).
*^2^* 1 = occurring predominantly in broadleaf evergreen forest; 2 = occurring mainly in non-broadleaf evergreen forest habitats. Classification following Fooden (1982a). Data were collated from Fooden (1982a), All The World's Primates (Rowe & Myers, 2011), and additional publications (*sources*; key to references can be found in Table S7).

**Table S9** Dietary ecology of macaques.

| **Species** | **Dietary breadth**  **(no. of types)** *^1^* | **% Fruits *^2^*** | **Range in**  **% fruits *^3^*** | **% Leaves *^2^*** | **Sources *^4^*** |
| --- | --- | --- | --- | --- | --- |
| *M. assamensis* | 8 | 40 | 53 | 39 | a, b, c, d, e, f |
| *M. cyclopis* | 9 | 51 | 8 | 27 | g |
| *M. fascicularis* | 7 | 74 | 23 | 9 | g, h, i |
| *M. fuscata* | 13 | 23 | 22 | 25 | g, j, k |
| *M. maura* | 6 | 71 | 10 | 8 | l |
| *M. mulatta* | 12 | 29 | 71 | 50 | m, n, o, p |
| *M. nemestrina* | 9 | 75 | 9 | 8 | g, i |
| *M. nigra* | 10 | 66 | 11 | 5 | q |
| *M. radiata* | 10 | 54 | 23 | 14 | i, r, s |
| *M. silenus* | 8 | 70 | 16 | 0 | s, t |
| *M. sinica* | 9 | 70 | 9 | 11 | i |
| *M. sylvanus* | 9 | 3 | 4 | 13 | g, j |

*^1^* Number of food types commonly included in the diet.
*^2^* Measured as the percentage of time spent eating fruits or leaves. When available, data were averaged across several populations to obtain species means.
*^3^* Constitutes the within-species range in the proportion of fruits in the diet, and denotes the difference between the minimum and maximum among reported population means.
*^4^* Data were collated from the Handbook of the Mammals of the World (Mittermeier, Rylands, & Wilson, 2013), All The World's Primates (2011), references therein, and the following additional publications: a) Heesen et al. (2013), b) Schülke et al. (2011), c) Huang et al. (2015), d) Kaewpanus et al. (2015), e) Koirala & Chalise (2014), f) Zhou et al. ( 2011), g) Ménard (2004), h) Yeager (1996), i) Rowe (1996), j) Hanya *et al.* (2011), k) Tsuji (2010), l) Sagnotti (2013), m) Richard *et al.* (1989), n) Sarker *et al.* (2008), o) Lindburg (1977), p) , Sengupta *et al.* (2014), q) O’Brien & Kinnaird (1997), r) Krishnamani (1994), s) Roy *et al.* (2012), t) Kumar (1987).

**Table S10** Mean male and female body mass of macaques.

| **Species** | **Males (kg)** | **Females (kg)** | **Sources** |
| --- | --- | --- | --- |
| *M. assamensis* | 11.2 | 7.3 | a |
| *M. cyclopis* | 6.0 | 4.9 | d |
| *M. fascicularis* | 5.5 | 3.6 | f |
| *M. fuscata* | 11.8 | 8.8 | g |
| *M. maura* | 8.0 | 5.6 | i |
| *M. mulatta* | 9.0 | 6.5 | j |
| *M. nemestrina* | 11.8 | 6.5 | k |
| *M. nigra* | 9.9 | 5.5 | i |
| *M. radiata* | 7.0 | 4.0 | l |
| *M. silenus* | 8.0 | 5.0 | k |
| *M. sinica* | 5.5 | 3.3 | n |
| *M. sylvanus* | 14.5 | 9.9 | o |

Data were collated from species accounts in the Handbook of the Mammals of the World (Mittermeier et al., 2013), Smith & Jungers (1997), and additional publications (*sources*; key to references can be found in Table S7).


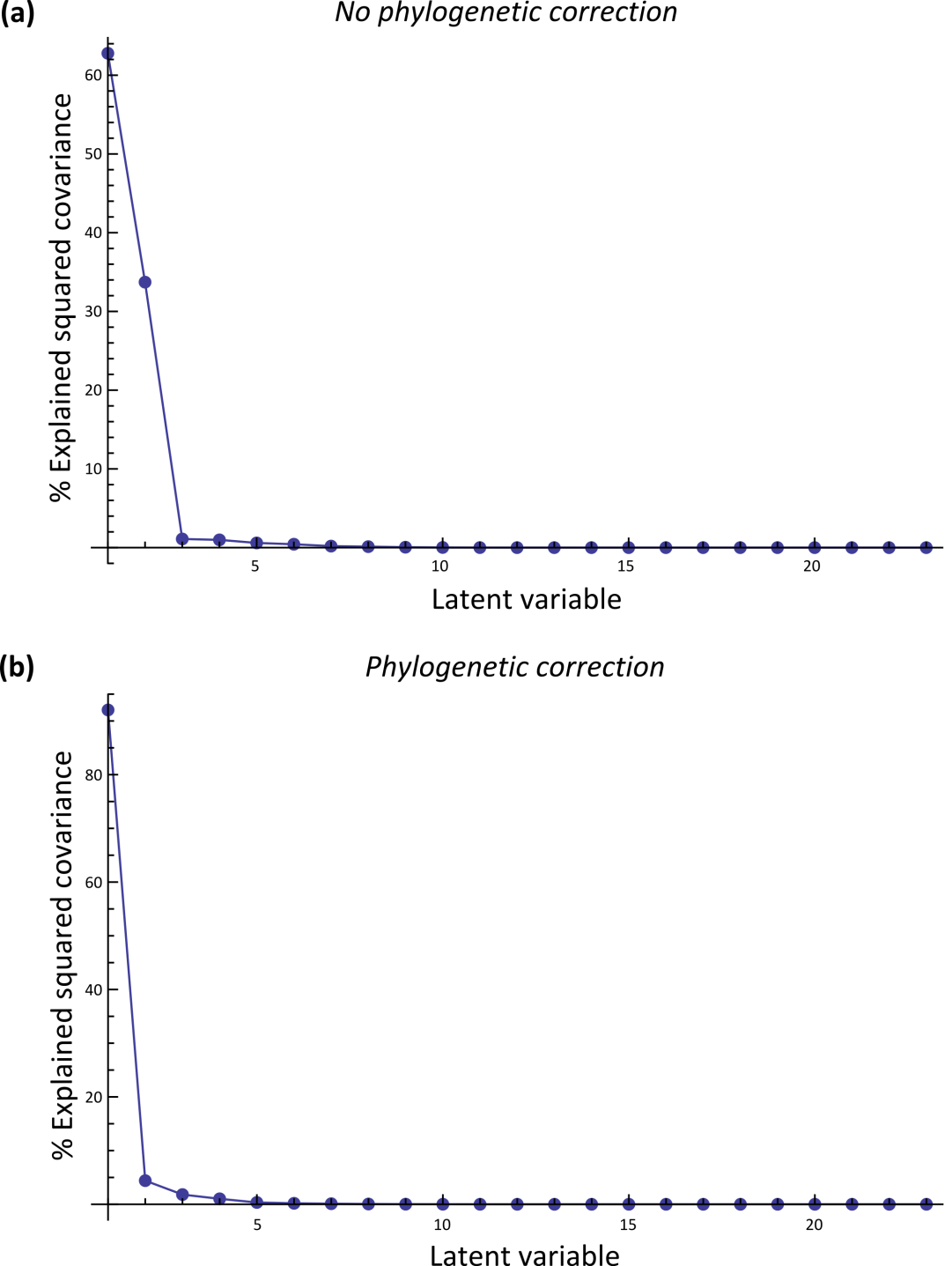


**Figure S2**. Scree plots of a) 2B-PLS without phylogenetic correction, and b) phylogenetic 2B-PLS. Latent variable 2 is diminished after phylogenetic correction.


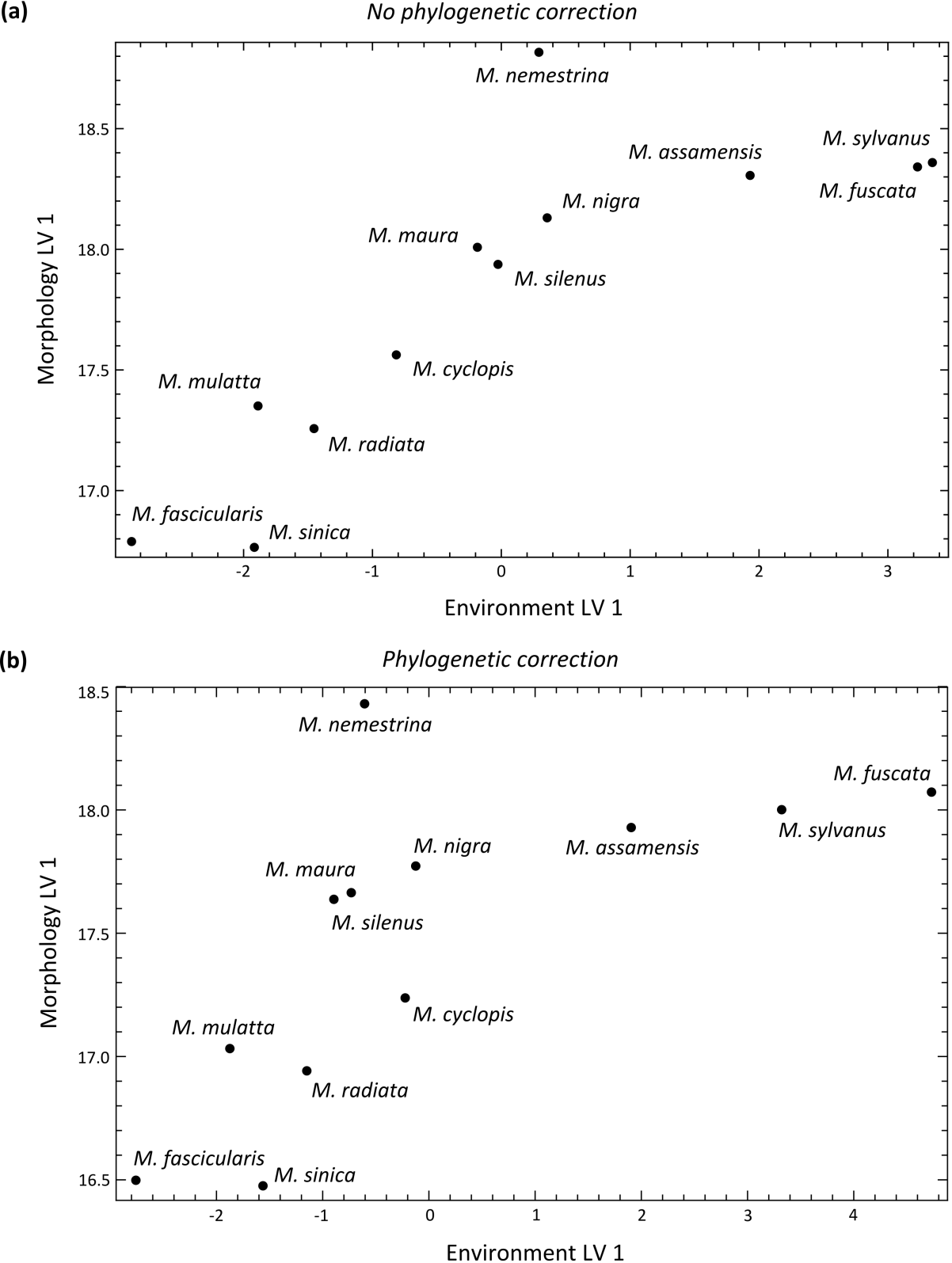


**Figure S3**. Scatter plots of partial least squares (PLS) scores on latent variable (LV) 1 for a) 2B-PLS without accounting for phylogeny, and b) phylogenetic 2B-PLS. In both cases, the covariance pattern between blocks shows a gradient in overall size: PLS scores vary from small-bodied species (e.g.,
*M. sinica* and *M. fascicularis*) to larger-bodied species (e.g., *M. sylvanus* and *M. fuscata*). See Table S10 for species' body masses.

**
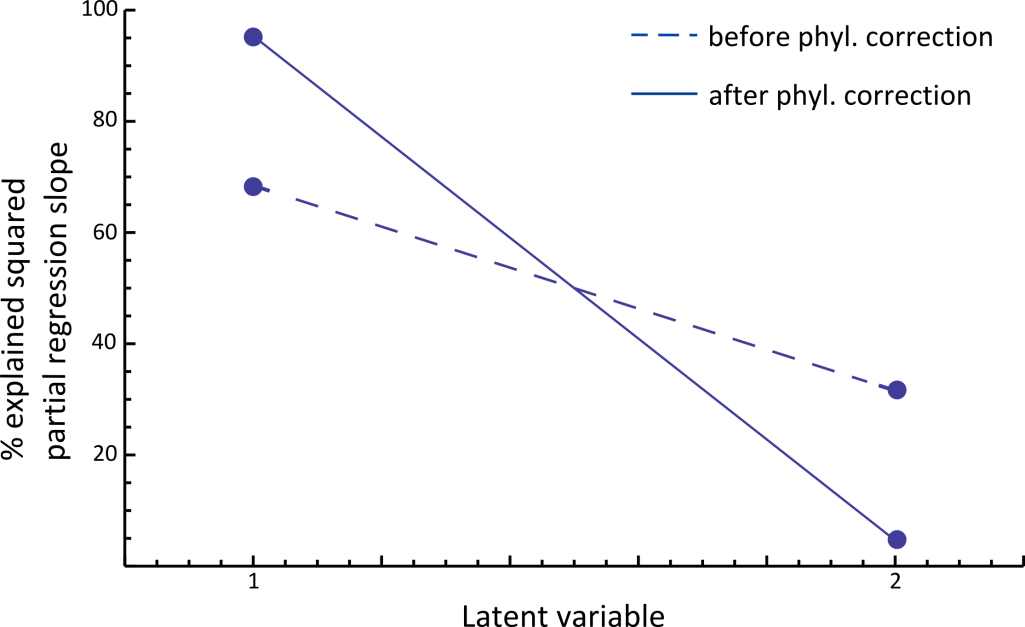
**

**Figure S4.** Scree plot of the geographic reduced rank regression before and after phylogenetic correction. Prior to phylogenetic correction, the reduced rank regression extracted two distinct latent variables (i.e., spatial gradients) that successively explain the maximum amount of phenotypic variation between macaques. Following phylogenetic correction, the first latent variable (LV 1) alone can explain nearly 100% of the phenotypic variation between macaques that is spatially structured.

**LITERATURE CITED**

Arnold, C., Matthews, L. J., & Nunn, C. L. (2010). The 10kTrees website: A new online resource for primate phylogeny. *Evolutionary Anthropology*, *19*(3), 114–118.

Fooden, J. (1969). *Taxonomy and Evolution of the Monkeys of Celebes*. *Bibliotheca Primatologica*. Basel, Switzerland: S. Karger.

Fooden, J. (1975). Taxonomy and Evolution of Liontail and Pigtail Macaques (Primates: Cercopithecidae). *Fieldiana Zoology*, *67*, 1–169.

Fooden, J. (1979). Taxonomy and Evolution of the Sinica Group of Macaques: I. Species and Subspecies Accounts of Macaca sinica. *Primates*, *20*(1), 109–140.

Fooden, J. (1981). Taxonomy and Evolution of the Sinica Group of Macaques: 2. Species and Subspecies Accounts of the Indian Bonnet Macaque, Macaca radiata. *Fieldiana Zoology*, *9*, 1–52.

Fooden, J. (1982). Ecogeographic Segregation of Macaque Species. *Primates*, *23*(4), 574–579.

Fooden, J. (1982). Taxonomy and Evolution of the Sinica Group of Macaques: 3. Species and Subspecies Accounts of Macaca assamensis. *Fieldiana Zoology*, *10*, 1–52.

Fooden, J. (1986). Taxonomy and Evolution of the Sinica Group of Macaques: 5. Overview of Natural History. *Fieldiana Zoology*, *29*, 1–22.

Fooden, J. (1988). Taxonomy and Evolution of the Sinica Group of Macaques: 6. Interspecific Comparisons and Synthesis. *Fieldiana Zoology*, *45*, 1–44.

Fooden, J. (1996). Systematic Review of Southeast Asian Longtail Macaques, *Macaca fascicularis* (Raffles, [1821]). *Fieldiana Zoology*, *81*, 1–206.

Fooden, J. (2000). Systematic Review of the Rhesus Macaque, *Macaca mulatta* (Zimmermann, 1780). *Fieldiana Zoology*, *69*, 1–180.

Fooden, J. (2006). Comparative Review of *Fascicularis*-Group Species of Macaques (Primates: *Macaca*). *Fieldiana Zoology*, *107*, 1–43.

Fooden, J. (2007). Systematic Review of the Barbary Macaque, *Macaca Sylvanus* (Linnaeus, 1758). *Fieldiana Zoology*, *113*, 1–58.

Fooden, J., & Aimi, M. (2005). Systematic Review of Japanese Macaques, *Macaca fuscata* (Gray, 1870). *Fieldiana Zoology*, *104*, 1–198.

Fooden, J., & Wu, H. Y. (2001). Systematic Review of the Taiwanese Macaque, *Macaca cyclopis* Swinhoe, 1863. *Fieldiana Zoology*, *98*, 1–69.

Hanya, G., Ménard, N., Qarro, M., Ibn Tattou, M., Fuse, M., Vallet, D., … Wada, K. (2011). Dietary adaptations of temperate primates: comparisons of Japanese and Barbary macaques. *Primates*, *52*(2), 187–98.

Heesen, M., Rogahn, S., Ostner, J., & Schülke, O. (2013). Food abundance affects energy intake and reproduction in frugivorous female Assamese macaques. *Behavioral Ecology and Sociobiology*, *67*(7), 1053–1066.

Hijmans, R. J., Cameron, S. E., Parra, J. L., Jones, P. G., & Jarvis, A. (2005). Very high resolution interpolated climate surfaces for global land areas. *International Journal of Climatology*, *25*, 1965–1978.

Huang, Z., Huang, C., Tang, C., Huang, L., Tang, H., Ma, G., & Zhou, Q. (2015). Dietary adaptations of Assamese macaques (*Macaca assamensis*) in limestone forests in Southwest China. *American Journal of Primatology*, *77*(2), 171–185.

IUCN Red List. (2007). IUCN Habitats Classification Scheme. IUCN Red List of Threatened Species. Retrieved from http://www.iucnredlist.org/technical-documents/classification-schemes/habitats-classification-scheme-ver3

Jones, K. E., Bielby, J., Cardillo, M., Fritz, S. A., O’Dell, J., Orme, C. D. L., … Purvis, A. (2009). PanTHERIA: a species-level database of life history, ecology, and geography of extant and recently extinct mammals. *Ecology*, *90*(9), 2648–2648.

Kaewpanus, K., Aggimarangsee, N., Sitasuwan, N., & Wangpakapattanawong, P. (2015). Diet and Feeding Behavior of Assamese Macaques (*Macaca assamensis*) at Tham Pla Temple, Chaing Rai Province, Northern Thailand. *Journal of Wildlife in Thailand*, *22*(1).

Koirala, S., & Chalise, M. K. (2014). Feeding Ecology of Assamese Macaque (*Macaca assamensis*) in Nagarjun Forest of Shivapuri Nagarjun National Park, Nepal. *Nepalese Journal of Zoology*, *2*(1), 31–38.

Krishnamani, R. (1994). Diet composition of the Bonnet macaque (*Macaca radiata*) in a tropical dry evergreen forest of southern India. *Tropical Biodiversity*, *2*(2), 285–302.

Kumar, A. (1987). *The ecology and population dynamics of the lion-tailed macaque (*Macaca silenus*) in South India*. University of Cambridge, Cambridge.

Lindburg, D. G. (1977). Feeding behaviour and diet of rhesus monkeys (*Macaca mulatta*) in a Siwalik Forest in North India. In T. H. Clutton-Brock (Ed.), *Primate Ecology: Studies of Feeding and Ranging Behaviour in Lemurs, Monkeys, and Apes* (pp. 223–249). London: Academic Press.

Ménard, N. (2004). Do ecological factors explain variation in social organization? In B. Thierry, S. M., & W. Kaumanns (Eds.), *Macaque Societies: A Model for the Study of Social Organization* (pp. 237–262). Cambridge: Cambridge University Press.

Mittermeier, R. A., Rylands, A. B., & Wilson, D. E. (Eds.). (2013). *Handbook of the Mammals of the World. Vol. 3. Primates*. Barcelona: Lynx Edicions.

Molur, S., Brandon-Jones, D., Dittus, W., Eudey, A. A., Kumar, A., Singh, M., … Walker, S. (2003). *Status of South Asian Primates: Conservation Assessment and Management Plan Report. Workshop Report, 2003*. Coimbatore, India.

Muroyama, Y., & Yamada, A. (2010). Conservation: present status of the Japanese macaque population and its habitat. In N. Nakagawa, M. Nakamichi, & H. Sugiura (Eds.), *The Japanese Macaques* (pp. 143–164). Tokyo: Springer.

O’Brien, T. G., & Kinnaird, M. F. (1997). Behavior, Diet, and Movements of the Sulawesi Crested Black Macaque (*Macaca nigra*). *International Journal of Primatology*, *18*(3), 321–351.

Richard, A. F., Goldstein, S. J., & Dewar, R. E. (1989). Weed Macaques: The Evolutionary Implications of Macaque Feeding Ecology. *International Journal of Primatology*, *10*(6), 569–594.

Rowe, N. (1996). *The Pictorial Guide to the Living Primates*. East Hampton, NY: Pogonias Press.

Rowe, N., & Myers, M. (Eds.). (2011). *All the World’s Primates, www.alltheworldsprimates.org*. Charlestown, RI: Primate Conservation Inc. Retrieved from www.alltheworldsprimates.org

Roy, K., Singh, M., & Singh, M. (2012). Diet and dietary-niche breadth of diurnal rain forest primates in the central western Ghats, India. *Folia Primatologica*, *82*(6), 283–298.

Sagnotti, C. (2013). *Diet Preferences and Habitat Use in Relation to Reproductive States in Females of a Wild Group of* Macaca maura *Inhabiting Karaenta Forest, South Sulawesi*. Hasanuddin University, Makassar.

Sarker, G. C., Kabir, M. M., Feeroz, M. M., & Hasan, M. K. (2008). Food and feeding behaviour of rhesus macaque (*Macaca mulatta*) at Barmi, Gazipur, Bangladesh. *Bangladesh Journal of Life Sciences*, *20*(2), 1–8.

Schülke, O., Pesek, D., Whitman, B. J., & Ostner, J. (2011). Ecology of Assamese macaques (*Macaca assamensis*) at Phu Khieo Wildlife Sanctuary, Thailand. *Journal of Wildlife in Thailand*, *18*, 1–15.

Sengupta, A., McConkey, K. R., & Radhakrishna, S. (2014). Seed Dispersal by Rhesus Macaques *Macaca mulatta* in Northern India. *American Journal of Primatology*, *76*(12), 1175–84.

Smith, R. J., & Jungers, W. L. (1997). Body mass in comparative primatology. *Journal of Human Evolution*, *32*(6), 523–59.

Tsuji, Y. (2010). Regional, Temporal, and Interindividual Variation in the Feeding Ecology of Japanese Macaques. In N. Nakagawa, M. Nakamichi, & H. Sugiura (Eds.), *The Japanese Macaques* (pp. 99–127). Tokyo: Springer.

Yeager, C. P. (1996). Feeding ecology of the long-tailed macaque (*Macaca fascicularis*) in Kalimantan Tengah, Indonesia. *International Journal of Primatology*, *17*(1), 51–62.

Zhou, Q., Wei, H., Huang, Z., & Huang, C. (2011). Diet of the Assamese macaque *Macaca assamensis* in limestone habitats of Nonggang, China. *Current Zoology*, *57*(1), 18–25.
